# Artificial Intelligence, Multi-Omics, and Multimodal Integration in Precision Radiotherapy for Head and Neck Cancer: From Biological Insights to Treatment Decisions

**DOI:** 10.3390/biomedicines14102252

**Published:** 2026-10-05

**Authors:** Miao Rao, Yingjian Wang, Minglun Li, Run Shi

**Affiliations:** 1 Guangxi Medical University Cancer Hospital, Nanning 530000, China; 2 Department of Urology, The First Affiliated Hospital of Zhengzhou University, Zhengzhou 450052, China; 3 Department of Radiation Oncology, Klinikum Lüneburg, 21339 Lüneburg, Germany; 4 Brain Metastases Diagnosis and Treatment Center, The Affiliated Brain Hospital of Nanjing Medical University, Nanjing 210029, China

**Keywords:** artificial intelligence, multi-omics, multimodal integration, head and neck cancer, precision radiotherapy, biomarkers, treatment selection, clinical validation

## Abstract

Radiotherapy for head and neck cancer must balance disease control against normal-tissue injury. Artificial intelligence (AI), multi-omics, and multimodal analysis offer complementary ways to characterize tumor and host heterogeneity, but their relevance to treatment decisions depends on how measurements, outcomes, and validation are linked. This narrative review examines these approaches across head and neck cancers, with evidence concentrated in head and neck squamous cell carcinoma and nasopharyngeal carcinoma. Literature was identified through Web of Science and PubMed searches with targeted supplementary searches and reference-list screening, and selected for relevance to radiotherapy biology, clinical outcomes, and methodological or clinical validation. We distinguish joint molecular or multimodal inputs from parallel analyses and post hoc biological annotation, and assess findings according to population, treatment setting, sampling time, and analytical unit. Genomic, proteogenomic, single-cell, and spatial studies identify candidate phenotypes involving deoxyribonucleic acid (DNA) repair, viral regulation, immunity, and metabolism. Clinical studies examine response, recurrence and survival stratification, molecular surveillance, and toxicity prediction. However, associations with outcomes under observed treatment do not establish differential treatment benefit, and improved discrimination does not demonstrate that model-guided care improves patient outcomes. Small patient cohorts, incomplete assay availability, potential data leakage, and limited independent validation further constrain interpretation. Translation requires reproducible measurements, patient-level validation of complete analytical pipelines, calibrated risk estimates, and evaluation within the intended treatment context. Prospective evaluation should prioritize predefined model-guided strategies and assess disease control, patient-relevant harm, and practical implementation together.

## 1. Introduction

Radiotherapy is central to head and neck cancer treatment, where precision therapy must balance disease control against normal-tissue damage. This balance varies across anatomical sites, viral etiologies, and treatment settings: genomic studies demonstrate substantial heterogeneity within head and neck squamous cell carcinoma (HNSCC), while nasopharyngeal carcinoma (NPC) has distinct molecular and immune features [1,2,3]. In a trial-treated oropharyngeal population, human papillomavirus (HPV) status retained an independent association with survival after adjustment for clinical factors, while tobacco exposure and stage further separated risk [4]. The clinical value of individualization also depends on the treatment being considered: gemcitabine–cisplatin induction improved overall survival before concurrent chemoradiotherapy in locoregionally advanced NPC [5], whereas PARSPORT demonstrated that parotid-sparing intensity-modulated radiotherapy (IMRT) could reduce xerostomia compared with conventional treatment [6]. These interventions improve the therapeutic balance through better cancer control or reduced normal-tissue exposure; they do not establish a single biomarker-guided strategy. Differences in tumor biology and host sensitivity may help refine risk assessment but limit biomarker transfer between populations and endpoints [7,8]. Precision therapy therefore requires a link between patient characteristics and a defined clinical question, such as recurrence risk, radiation toxicity, or the relative benefit of alternative treatments.

Artificial intelligence (AI) offers methods for analyzing the complementary information generated by genomics, transcriptomics, epigenomics, proteomics, metabolomics, and emerging single-cell and spatial technologies. Integrating these measurements with imaging, pathology, dose distributions, and clinical data may help characterize disease phenotypes and improve prediction, but integration must be clearly defined [9,10]. Molecular multi-omics, imaging–dose fusion, and biological annotation of an imaging model represent different analytical designs; parallel measurements do not necessarily enter a predictor together [7,11]. Likewise, molecular subtypes and cellular states can provide biological hypotheses without having been validated as markers of radiotherapy response [2,3]. Proteogenomic profiling of HPV-negative HNSCC has exposed signaling and immune features not fully represented by genomic alterations alone [12], and single-cell analysis has localized malignant-cell programs within heterogeneous tumor ecosystems [13]. These measurements offer complementary biological information, but adding variables does not necessarily improve prediction. The value of AI depends on how measurements are linked within patients and whether they address a clinically meaningful endpoint.

Evidence of predictive performance currently exceeds evidence that these tools improve care. A systematic review of machine-learning models for radiation-induced toxicity in head and neck cancer found frequent high risk of bias, limited calibration reporting, and no studies demonstrating clinical implementation or clinical utility within its included evidence [14]. Discrimination alone cannot establish the accuracy of predicted risks, transportability to another population, or the value of acting on a model output [15,16]. The distinction is particularly consequential for treatment de-intensification: in HPV-positive oropharyngeal cancer, trials replacing cisplatin with cetuximab showed inferior cancer outcomes despite favorable underlying prognosis [17,18]. The required evidence therefore depends on the decision: prognostic classification alone does not show that a particular treatment change preserves control or reduces harm.

Existing reviews have organized radiomics, radiogenomics, and machine-learning applications by workflow or clinical endpoint and emphasized reproducibility and external validation [9,10]. These reviews help assess how biological information can inform treatment, but gaps remain between molecular characterization, validated prediction, and demonstrated clinical utility [10,14]. A synthesis organized around these distinctions can separate findings on disease biology, outcomes under observed care, and treatment choices. Promising results can then be assessed against their data requirements, validation limits, and relevance to the balance between tumor control and toxicity.

**Figure 1:**
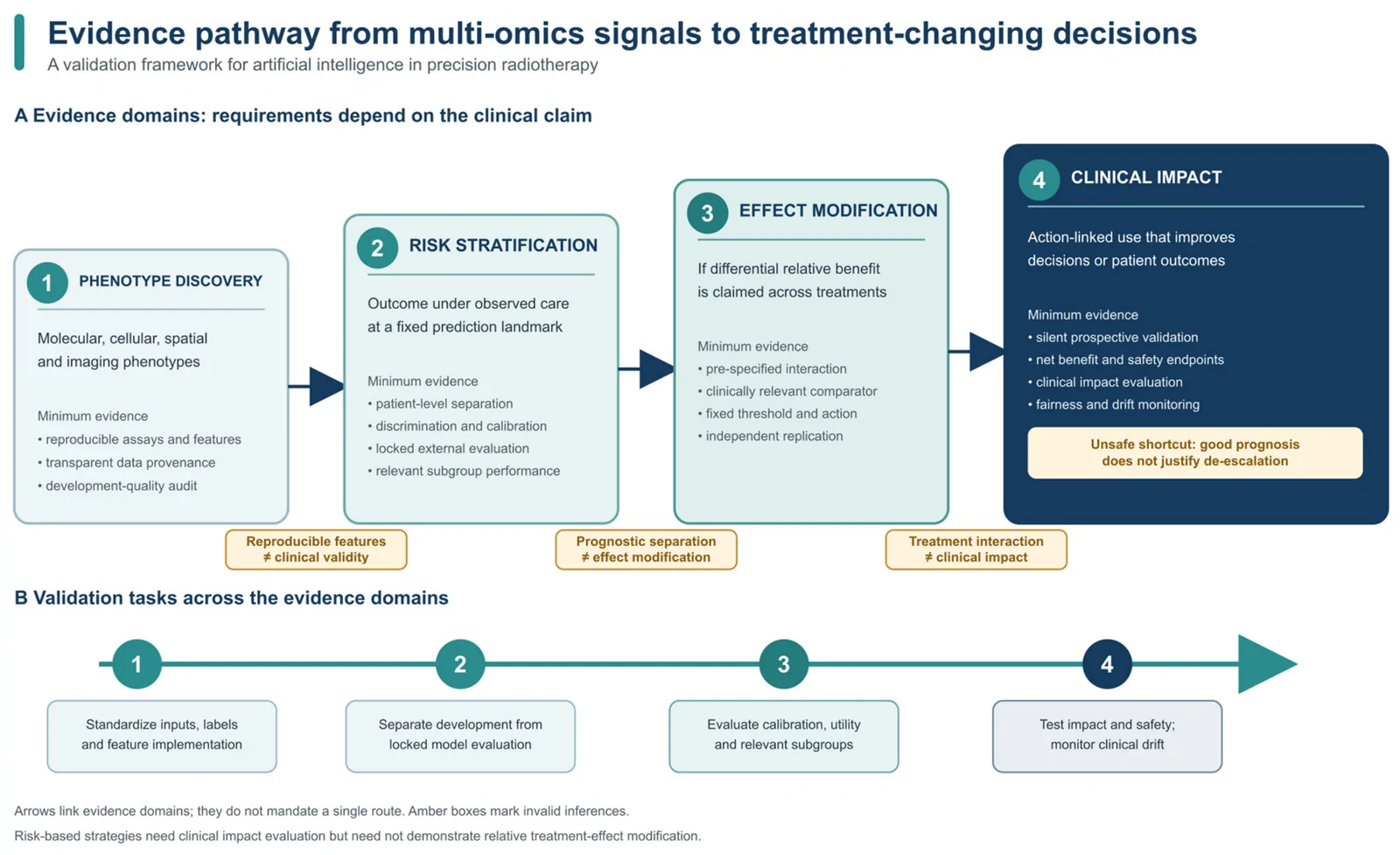
From multi-omics signals to treatment-changing evidence in precision radiotherapy. (**A**) The framework distinguishes biological phenotype discovery, risk stratification under observed care, relative treatment-effect modification, and clinical impact. Reproducible feature computation does not establish clinical validity [19], prognostic separation does not establish relative treatment benefit [20], and a treatment interaction does not by itself demonstrate improved patient outcomes [15,16]. Randomized evidence in HPV-positive oropharyngeal cancer illustrates that favorable prognosis cannot directly justify treatment substitution [18]. (**B**) The validation backbone links standardized inputs and feature implementation to separation of development from locked evaluation, calibration and subgroup assessment, and prospective impact evaluation [15,16,19]. Solid arrows link evidence domains rather than mandate a single validation route; amber boxes denote invalid inference shortcuts. The interaction domain applies when differential relative benefit is claimed. Risk-based treatment strategies still require clinical impact evaluation, but not necessarily relative treatment-effect modification. This is an original synthesis. AI, artificial intelligence; HPV, human papillomavirus; RT, radiotherapy. The framework is author-developed and has not itself been empirically validated; its arrows organize evidentiary requirements rather than demonstrate tested transitions.

This narrative review examines AI-enabled multi-omics and related multimodal approaches to precision radiotherapy across head and neck cancers. The evidence is concentrated in HNSCC and NPC, so conclusions should remain specific to the studied populations rather than extend to less-represented histologies. We address three review questions: (1) What constitutes meaningful multi-omics or multimodal integration, as distinct from parallel analysis or post hoc annotation? (2) What evidence connects these approaches to tumor biology, treatment response, recurrence, survival, and normal-tissue toxicity? (3) What validation is needed before the findings can inform treatment selection or modification while balancing disease control against patient-relevant harm? Figure 1 organizes phenotype discovery, risk stratification, treatment-effect modification, and clinical impact as distinct evidence domains with claim-specific validation requirements [15,16,19]. Its arrows link these domains without implying biological causality or requiring every risk-based strategy to demonstrate relative treatment-effect modification.

## 2. Review Approach and Literature Selection

We conducted a narrative review of biological, predictive, and treatment-decision evidence. Web of Science Core Collection and PubMed were searched on 8 September 2026 using four concept blocks: head and neck cancer, radiotherapy, molecular omics, and artificial intelligence or multimodal analysis. The saved database-specific queries, including field tags and synonyms, are reproduced in Supplementary Methods S1; no date or language restriction was specified in these initial queries. Bibliographic identifiers and titles were used for deduplication. Title and abstract screening prioritized studies of radiation response, disease control, toxicity, or integration and validation relevant to radiotherapy. Studies outside the disease scope and diagnostic, surgical, or drug-target reports without a relevant radiotherapy connection were excluded from the initial core set. Readable full texts were then assessed for their contribution to the review questions.

Additional relevant literature was identified through targeted supplementary searches and reference-list screening. The targeted PubMed supplementation on 12 September 2026 used topic searches, focused title searches, and identifier lookups. Supplementary Methods S1 provides the initial database-specific queries, selected supplementary topic queries, and selection criteria. The 109 references include primary studies, background and clinical-context sources, and methodological publications; they do not represent 109 independent clinical cohorts. Table 1 separates retrieval stages; Table 2 groups selected primary studies by their role in the synthesis.

Full-text reports and available supplements were read for all cited references. A structured descriptive assessment of all studies contributing substantially to patient-level prediction or methodological conclusions recorded population and treatment, sampling time, independent analytical unit, endpoint, patient-level separation, external evaluation, calibration, model selection, possible leakage, and comparison with clinical or single-input baselines (Supplementary Table S2). Patient and event counts, confidence intervals, and threshold-dependent performance were retained where verified. Unconfirmed reporting was distinguished from a documented methodological limitation; this inventory is not a formal risk-of-bias score. Biological findings, prognostic associations, treatment interactions, and clinical impact were synthesized separately, without quantitative pooling.

## 3. Evidence Landscape and Integration Boundaries

We distinguish molecular multi-omics from integration of imaging, dose, pathology, or clinical information, and classify each application by its analytical objective and treatment setting. Clinical studies address patient outcomes, irradiation experiments test functional responses, and molecular or methodological studies provide biological or analytical context [2,7,21,22]. Direct evidence concerns AI, molecular, or multimodal analyses linked to radiation biology or patient outcomes. Conventional treatment trials and delivery-oriented AI studies are considered separately as clinical context for judging the consequences of a treatment decision.

### 3.1. Joint Inputs, Parallel Analysis, and Post Hoc Annotation

Molecular multi-omics integrates at least two distinct molecular assay layers; multimodal integration combines different information types, such as molecular measurements, imaging, pathology, dose, or clinical variables. Single-cell and spatial analyses describe measurement resolution or organization, whereas circulating biomarkers are defined by their specimen source. None constitutes multi-omics by itself. Joint-input analyses combine linked measurements through features, shared representations, or model outputs [22,23,24]. Parallel analyses remain separate, whereas post hoc annotation uses another data source to interpret an established predictor. Magnetic resonance imaging (MRI) plus dose features therefore represent imaging–dose fusion [11]; transcriptomic interpretation of an MRI score is post hoc annotation when transcripts do not enter the predictor [7]. Here, radiomics or clinical-only models are included as comparators or clinical-context evidence, not relabeled as molecular multi-omics.

The incremental value of an added modality should be assessed in comparable patients, using the same outcome and a consistent evaluation pipeline, against clinical and single-modality baselines; feature importance alone does not establish added predictive value [25]. Validation must include feature selection and tuning so that comparisons reflect the complete modeling pipeline, rather than differences in model selection or evaluation [15]. Prediction time should be explicit: models incorporating post-treatment imaging address a later prediction task and cannot be interpreted as pretreatment decision tools [26].

### 3.2. Integration Methods and Analytical Objectives

Supervision describes the learning objective, whereas graph-based modeling describes a representation; the two are not mutually exclusive. Data Integration Analysis for Biomarker discovery using Latent cOmponents (DIABLO) integrates cross-omics features for supervised class discrimination [23]. Multi-Omics Factor Analysis (MOFA) and MOFA+ learn unsupervised factors representing shared and assay-specific variation [22,27]. Multi-Omics Graph cOnvolutional NETworks (MOGONET) performs supervised classification using omics-specific sample graphs [24], whereas similarity network fusion combines sample-similarity networks for unsupervised structure discovery [28]. Weighted-nearest-neighbor analysis integrates paired modalities at the cell level [29]. Supplementary Table S1 compares their objectives, inputs, missing-data handling, interpretability, and principal limitations.

Method choice should follow the analytical question, available inputs, and planned validation. Explained variance and classification accuracy measure different objectives; neither demonstrates clinical utility in radiotherapy [22,24]. Missing-data support, preprocessing choices, and representation stability must be assessed within the intended application rather than assumed from the method name [22,27]. Comparisons should therefore preserve the prediction target and validation design, including whether feature selection and tuning were confined to development data.

### 3.3. Populations, Disease Subtypes, and Treatment Settings

The synthesis separates non-nasopharyngeal HNSCC from NPC and retains site, viral status, and treatment history within each group. The cited EBV-associated NPC atlases describe regulatory and immune heterogeneity, not validated radiotherapy-response predictors [2,3]. Low-risk p16-positive oropharyngeal cancer studied for initial-treatment de-intensification is not interchangeable with recurrent NPC receiving re-irradiation [7,30]. Within HNSCC, HPV-driven and carcinogen-driven tumors also differ in immune programs [31]. Findings are therefore interpreted within the reported anatomical site, viral definition, stage, and regimen, rather than generalized to all head and neck cancers.

Clinical sample size is defined by independent patients, not by their images, cells, spatial regions, or repeated samples. Training and evaluation data should be separated by patient before data-adaptive processing, with augmentation restricted to development data [15]. Molecular associations, in vitro irradiation responses, and patient-level treatment interactions represent different inferential targets [20,21]. Re-irradiation necrosis, primary-treatment disease control, and combined-treatment response cannot be ranked as equivalent endpoints; poor transfer between systemic regimens alone does not establish treatment-predictive specificity [7,32].

## 4. Biological Phenotypes and Radiotherapy Relevance

### 4.1. Genomic and DNA Damage-Repair Phenotypes

Genomic profiling identifies biological differences whose functional consequences require testing. The Cancer Genome Atlas (TCGA) characterized HPV-negative HNSCC by frequent tumor protein p53 (TP53) and cyclin-dependent kinase inhibitor 2A (CDKN2A) inactivation and extensive copy-number alterations, alongside recurrent alterations involving chromatin regulation, differentiation, and growth signaling [1]. These findings provide candidate features. DeepFoci instead automates quantification of radiation-induced deoxyribonucleic acid (DNA) damage foci in microscopy images. Its agreement with two expert annotations yielded Dice coefficients of 0.64 and 0.70, compared with 0.75 between experts [33]. These results evaluate a functional-assay measurement tool, not patient-level radiosensitivity prediction or molecular multi-omics integration. Ataxia telangiectasia and Rad3-related protein (ATR) perturbation further separates molecular abundance from radiation response. In the tested HNSCC cell lines, lower ATR protein abundance in HPV-positive cells did not correspond to impaired normalized ATR activation, while small interfering ribonucleic acid (siRNA)-mediated ATR depletion increased radiosensitivity predominantly in the HPV-positive models [21]. The authors concluded that reduced ATR abundance alone did not explain their inherent radiosensitivity [21]. A model using expression as a proxy for repair capacity may therefore capture a different phenotype from one based on functional measurements or perturbation responses.

Proteogenomics can test whether genomic or transcriptional changes accompany altered signaling activity. In 108 HPV-negative HNSCCs, integrated protein and phosphosite measurements revealed distinct modes of epidermal growth factor receptor (EGFR) activation, immune-associated alterations, and molecular subtypes with proposed therapeutic vulnerabilities [12]. This supports using protein-level information to refine target hypotheses rather than assuming that an altered gene has a uniform functional consequence. A separate analysis integrating existing single-cell, bulk and deconvolution data associated inferred HNSCC radioresistance with cell-cycle, metabolic, hypoxic and immune programs [34]. These computational associations can prioritize pathways for testing, but they do not show that every profiled cell was experimentally irradiated or that a patient will benefit from inhibiting the nominated pathway. A radiation-focused integrative model should specify its biological target: mutation status, pathway activity, inferred sensitivity and measured survival after irradiation are related but distinct.

Associations with patient outcomes also depend on the biological context of the repair phenotype. In the Netherlands Cancer Institute (NKI) chemoradiotherapy cohort, predicted DNA-repair deficiency was not significantly associated with overall survival across all patients, but was associated with poorer survival in the gene-signature-defined normoxic subgroup (hazard ratio (HR), 2.83; 95% confidence interval (CI), 1.36–5.86); the hypoxic subgroup showed no significant association [35]. The association with distant metastasis in the normoxic subgroup was significant in NKI but did not reach statistical significance in the independent DESIGN cohort, where the confidence interval was wide [35]. Whether repair deficiency relates consistently to clinical outcome, or marks a differential benefit from radiotherapy, remains open on this evidence.

Target selection requires distinguishing a gene’s contribution to tumor growth from its effect on irradiation response. In a tumor-doubling-time-related signature study, epithelial cell transforming 2 (ECT2) knockdown reduced CAL27 proliferation, DNA synthesis, and colony formation [36]. These experiments support a growth-related function, but the corresponding assays did not test whether ECT2 suppression changes radiation response. By contrast, the NPC study of the neddylation inhibitor MLN4924 directly tested a treatment combination: after irradiation, treated HNE1 and HNE2 cells formed more surviving colonies than irradiation controls, and the combined treatment produced larger tumors in the HNE1 xenograft model [37]. An intervention selected through molecular analysis can therefore interact unfavorably with irradiation even when its target appears relevant to tumor biology. This model-specific result does not establish a clinical contraindication or a universal effect of NEDD8-activating enzyme E1 subunit 1 (NAE1) inhibition, but it makes combination testing essential to the proposed radiosensitization claim. AI-based target prioritization should keep proliferation phenotypes, radiation-response phenotypes, and patient outcome associations separate until experiments connect them under defined dose and scheduling conditions.

Functional models allow candidate interventions to be tested before patient treatment. An HNSCC organoid platform retained tumor-associated characteristics and showed heterogeneous responses to drugs and irradiation in vitro [38]. Its potential depends on establishing clinically representative samples quickly enough: a later feasibility study obtained organoids from 35% of patient tumors overall, rising to 77% among samples with at least 30% tumor cells, while prior radiotherapy and processing delays were negative predictors of establishment [39]. Patients with successful cultures may therefore not represent all those needing a treatment decision. Direct combination experiments also provide evidence that expression-based predictions cannot supply alone. Polo-like kinase 1 (PLK1) inhibition with onvansertib reduced colony formation after irradiation in HPV-negative HNSCC models and reduced tumor growth in vivo, with ribonucleic acid (RNA) sequencing and knockdown analyses implicating matrix metallopeptidase 10 (MMP10)-related regulation [40]. This contrasts with the unfavorable irradiation interaction reported for MLN4924 in the NPC models discussed above [37]. The comparison separates an antiproliferative drug from a radiosensitizer as labels; comparative drug efficacy across these different experimental systems remains untested.

### 4.2. EBV-Associated Epigenomes and NPC Cellular Ecology

**Figure 2:**
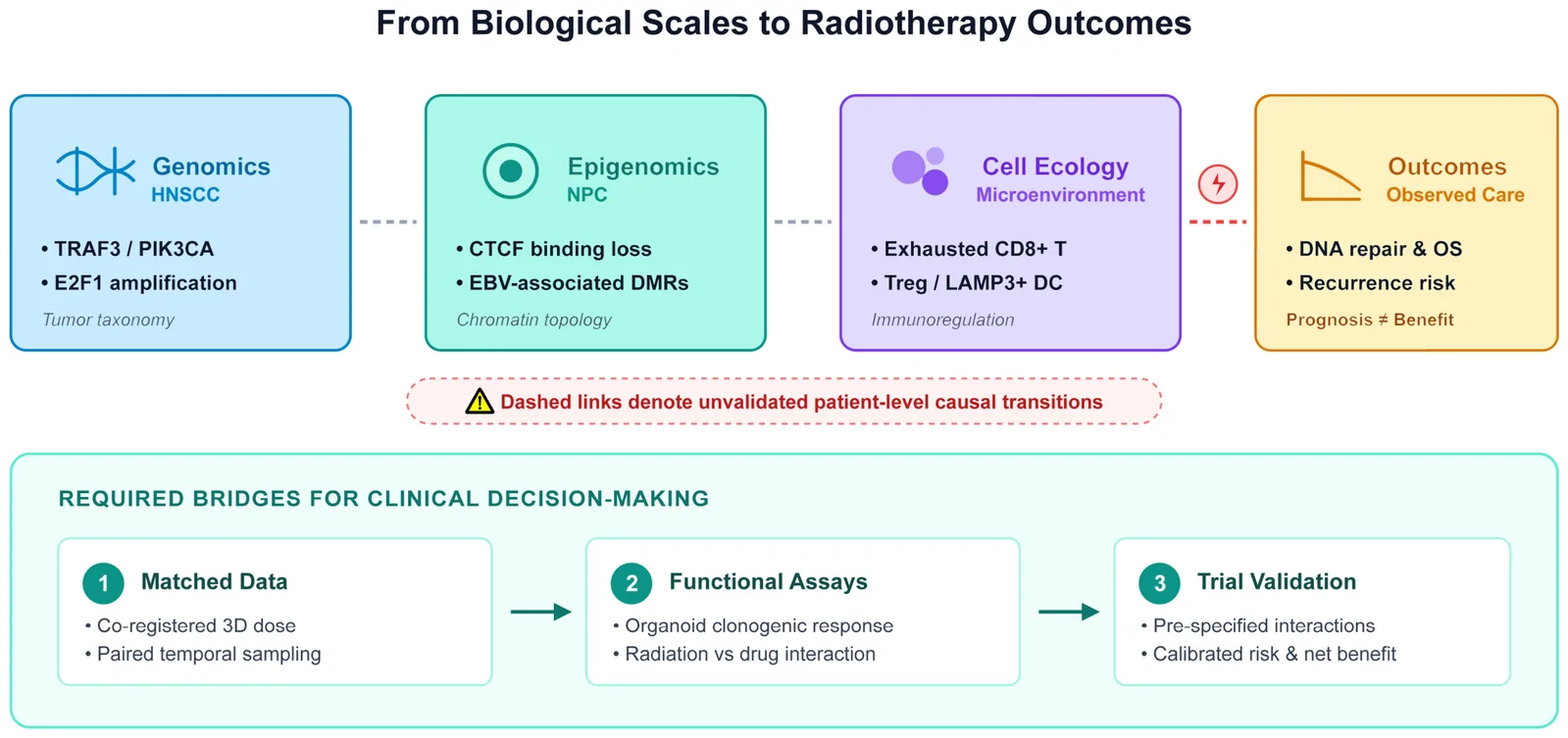
From molecular phenotypes to radiotherapy outcomes: missing bridges across biological scales. The four evidence boxes summarize HPV-associated HNSCC genomic features [1], EBV-associated NPC epigenomic dysregulation [2], NPC immune-cell states [3], and conditional associations between DNA-repair phenotypes and survival [35], respectively. Each box represents observations at its own measurement scale. Dashed arrows identify unvalidated transitions rather than an established patient-level causal pathway. Matched multi-omics measurements, functional perturbation, and clinical evaluation are proposed requirements for connecting these levels, not completed findings. This is an original synthesis. DC, dendritic cell; DMR, differentially methylated region; EBV, Epstein–Barr virus; HNSCC, head and neck squamous cell carcinoma; HPV, human papillomavirus; NPC, nasopharyngeal carcinoma; OS, overall survival; Treg, regulatory T cell. Additional terms: CD8, cluster of differentiation 8; CTCF, CCCTC-binding factor; DNA, deoxyribonucleic acid; E2F1, E2F transcription factor 1; LAMP3, lysosome-associated membrane protein 3; PIK3CA, phosphatidylinositol-4,5-bisphosphate 3-kinase catalytic subunit alpha; TRAF3, tumor necrosis factor receptor-associated factor 3. This author-developed conceptual framework is not an empirically validated model.

In NPC, epigenomic integration complements genomic profiling by characterizing regulatory heterogeneity. Combined methylation, chromatin-accessibility, exome, and single-cell analyses identified hypermethylated and hypomethylated NPC subtypes and linked EBV-associated differentially methylated regions to loss of CCCTC-binding factor (CTCF) binding [2]. This supports a relationship between viral context and chromatin dysregulation, but the subtypes’ prognostic and radiotherapy-predictive value remained unestablished [2]. Single-cell profiling resolves this heterogeneity at the cellular level. In untreated EBV-positive NPC, transcriptomic and T-cell receptor analyses identified programmed cell death 1 (PDCD1)-high exhausted CD8+ T-cell states, while tumor necrosis factor receptor superfamily member 4-positive (TNFRSF4+) regulatory T cells and lysosome-associated membrane protein 3-positive (LAMP3+) dendritic cells exhibited expression programs consistent with immunoregulation [3]. These observations characterize cellular states; their value as predictors of radiotherapy response is unproven [3]. Figure 2 places these findings alongside genomic, functional, and outcome evidence at their respective measurement scales. Dashed links mark unvalidated transitions; a causal sequence connecting CTCF abnormalities, immune-cell states, and repair-associated outcomes in the same patients has yet to be shown.

Other NPC atlases show why immune abundance alone is an incomplete representation. Single-cell analysis of 15 primary tumors identified immune subtype signatures associated with survival, including macrophage, dendritic-cell, natural-killer-cell and plasma-cell programs [41]. Another study profiled 19 EBV-positive tumors and identified malignant cells with an epithelial–immune expression pattern associated with poor prognosis; functional analyses supported their capacity to suppress T-cell interferon-γ production [42]. Single-cell, single-nucleus and imaging analyses further described immune-escape patterns involving loss of major histocompatibility complex molecules, epithelial–mesenchymal features and protective tumor-nest organization [43]. Together, these studies indicate that cell identity, state and neighborhood can convey different information. For radiotherapy, these studies supply candidate features and mechanisms; a validated immune score for selecting irradiation intensity has yet to emerge from them.

Spatially resolved comparisons highlight the relevance of treatment history. In 39 tumors from 24 patients, a primary-versus-recurrent NPC study reported enrichment of melanoma cell adhesion molecule-positive (MCAM+) cancer-associated fibroblasts in recurrent disease and a mechanism involving collagen IV, integrin subunit alpha 2 (ITGA2), focal adhesion kinase (FAK), and protein kinase B (AKT) promoting radioresistance, alongside impaired T-cell infiltration [44]. In a separate NPC study, tertiary lymphoid structure-associated populations and their interactions with tumor aggregates were linked to prognosis and programmed cell death protein 1 (PD-1) blockade response [45]. An association between an organized immune niche and immunotherapy response does not establish that the same niche predicts benefit from re-irradiation. These findings suggest that integration should retain spatial organization and prior-treatment status instead of reducing all immune measurements to a common high-versus-low infiltration variable. The recurrent tumor microenvironment is therefore better regarded as a distinct biological state than as a more advanced version of an untreated tumor.

A treatment-linked NPC study profiled 240 pretreatment tumors, with assay-specific availability, and defined three subtypes by proteomic clustering, subsequently characterized across molecular layers [46]. The subtypes differed in interferon signaling, cell-cycle activity, and immune exhaustion. Follow-up analyses connected immunoglobulin A-positive (IgA+) plasma cells to treatment response and evaluated tissue markers in three phase III trial cohorts [46]. The study extends beyond profiling untreated tumors to include formal treatment-interaction analyses. Proteomic subtyping, transcriptomic subtype prediction, and tissue-marker treatment interactions nonetheless remain separate analytical components; none amounts to a prospectively validated dose-selection algorithm.

### 4.3. Spatial Immunity, Metabolism, Microbiota, and Extracellular Vesicles

Spatial protein measurements reveal a layer of heterogeneity that a single patient-level score can obscure. In the matrix-assisted laser desorption/ionization mass-spectrometry imaging (MALDI-MSI) study, matching tissue peptide signals to tandem mass spectra and requiring concordant spatial distributions of at least two peptides per protein yielded twelve tentative protein identifications [32]. The corresponding peptide signals were lower in tumor regions from patients with recurrence or progression, while signal distributions also varied within individual tumors [32]. Because regions within a specimen can have different molecular profiles, biological interpretation must account for sampling and spatial aggregation. The maps support studying tissue heterogeneity and candidate protein associations; identifying radiation-resistant clones or deciding which subvolume should receive a higher dose lies beyond them.

Tissue composition can also affect the interpretation of a bulk signature. Single-cell analysis of primary and metastatic HNSCC localized a partial epithelial–mesenchymal transition (pEMT) program at the leading edge of primary tumors and linked it to nodal metastasis and adverse pathological features [13]. Multiparameter imaging subsequently connected the clinical associations of pEMT phenotypes to stromal composition, with spatial transcriptomics and spheroid experiments supporting a cancer-associated fibroblast–pEMT interaction [47]. Spatial arrangement itself carried prognostic information in a much smaller paired primary/recurrent HPV-negative HNSCC series: tumor–immune compartmentalization was associated with longer progression-free survival among nine patients [48]. These observations justify testing cell-state and neighborhood features in addition to total cell fractions. A radiation-resistant subvolume remains unidentified, and the small number of independent patients limits prognostic precision.

Hypoxia–immune classifications illustrate the potential and difficulty of adapting these phenotypes to routine assays. A 54-gene classifier separated HNSCC survival groups and was evaluated in two additional cohorts, with protein and multiplex-imaging observations providing complementary biological support [49]. In a different, 39-patient chemoradiotherapy cohort, a post hoc immunohistochemical classifier combining carbonic anhydrase IX and tumor-infiltrating lymphocytes separated locoregional-control outcomes; external validation remained necessary [50]. These studies support examining hypoxia and immunity together, but the tissue classifier should not be assumed to reproduce the transcriptomic classifier on a different platform. An implementable assay would need to preserve its own cutoffs, sampling regions and endpoint definition. Whether hypoxia modification or immune checkpoint inhibition improves outcomes in the classified groups requires its own treatment-comparison evidence.

Agreement across modalities in tissue and metabolic studies must be interpreted in relation to the measured target. A lactate-related RNA score was associated with poorer survival outcomes in HNSCC patients receiving adjuvant radiotherapy [51]. The accompanying pathology classifier estimated RNA-defined metabolic groups, while an external cohort showed higher lactate dehydrogenase A (LDHA) and monocarboxylate transporter 1 (MCT1) immunohistochemical scores in the extreme high-prediction group [51]. This offers cross-modal support for a tissue phenotype, but neither the RNA score nor protein staining directly measures lactate concentration; the pathology classification task also differs from predicting a radiotherapy outcome. Microbial and metabolite measurements provide complementary, but not automatically integrated, evidence. In an NPC study, nonresponders had higher acetate levels in tumor tissue and fecal samples, whereas propionate and butyrate did not show consistent between-group differences [52]. The machine-learning model used microbial abundance, with metabolite profiling performed in parallel [52]. The results identify response-associated candidates but establish neither a joint microbial–metabolic predictor nor a causal pathway from microbial composition through acetate to treatment resistance.

Vesicle-associated signals can also be measured without directly identifying their molecular cargo. A surface-enhanced Raman spectroscopy platform distinguished exosomes from radioresistant and parental NPC cells; its neural-network classifier reported a test area under the receiver operating characteristic curve (AUC) of 0.96 from a dataset of 1044 spectra [53]. The study developed a spectral measurement and classification workflow; it did not validate the method in 1044 patients or in patient-derived liquid biopsies. Internal calibration improved measurement repeatability, whereas protein profiling supplied a separate interpretive branch [53]. Stable spectra support analytical measurement, while protein associations suggest candidate explanations. Clinical prediction would still require independent biological batches and patient samples. Spectral separation therefore offers a route to assay development without demonstrating that a specific vesicle protein causes radioresistance.

Extracellular-vesicle studies distinguish cargo characterization from functional intervention. Proteomic screening of vesicles released by irradiated UM-SCC6 cells found nominal enrichment of DNA-repair-related proteins, but no significant enrichment of immune-related processes; the screen did not assess effects on recipient-cell function [54]. In a separate NPC xenograft study, adding the reported vesicle preparation in a radiotherapy–immunotherapy setting reduced tumor growth, with attenuation after phosphatase and tensin homolog (PTEN)-related knockdown [55]. The perturbation provides model-specific functional evidence, not proof that the screened vesicle cargo mediates clinical radioresistance. Across these studies, AI assisted measurement or phenotype estimation; whether this information improves patient stratification requires separate clinical prediction studies.

## 5. From Phenotypes to Clinical Stratification

### 5.1. Early Response, Recurrence, and Survival

Clinical stratification requires a specified outcome and prediction time. Table 2 distinguishes study design from inferential role: phenotype or prognostic association, formal treatment interaction, and randomized treatment comparison. Clinical utility requires evidence that using a defined model-guided strategy improves patient outcomes; neither decision-curve estimates nor a treatment trial without model-guided allocation establishes that utility [15,16]. Spatial proteomic profiles, for example, distinguished 24-month disease status after chemoradiotherapy [32], whereas a 12-gene signature estimated overall survival among irradiated TCGA patients (internal test AUCs, 0.661 at three years and 0.584 at five years) [56]. Both endpoints remain indirect measures of intrinsic radiosensitivity.

Biological classification and clinical prognosis also require separate validation. A computed tomography (CT)-based convolutional neural network (CNN) for spatial CD8+ immune phenotypes was externally evaluated using a chemokine-expression surrogate, whereas its clinical associations were examined in the ARTSCAN III oropharyngeal cancer cohort [66]. After adjustment for HPV/p16 status, stage, and treatment arm, the CNN-inflamed classification remained associated with local control and progression-free survival, but not significantly with overall survival [66]. Agreement with a molecular surrogate and association with a clinical endpoint support different aspects of the model; neither establishes equivalent performance across all outcomes. An alternative route uses tissue-based immune measurements: in resectable oral squamous cell carcinoma, a seven-feature immunohistochemical support vector machine (SVM) signature incorporated immune-cell densities in the tumor center and invasive margin, with survival associations reproduced in testing and validation cohorts [67]. This spatially defined assay was distinct from the same paper’s RNA-derived immune score, rather than a direct transfer of that score onto protein measurements. The assay supports the feasibility of measuring a specified tissue phenotype. Equivalence between RNA and immunohistochemical risk estimates would require a separate comparison.

Early-response studies in NPC illustrate how the assessment window changes the clinical question. A serum study proposed a seven-protein random-forest signature using 135 training and 164 validation patients, with response assessed six weeks after radiotherapy [59]. Its use of both tumor-area reduction and complete/non-complete response rules leaves the exact modeling label unclear [59]. A narrow-band imaging (NBI) study instead classified MRI response within three days of completing concurrent chemoradiotherapy in 57 patients; the classifier used pretreatment endoscopic images, while transcriptomic analyses supplied immune associations and cell-source annotation [62]. These studies identify candidate circulating and mucosal imaging signals for distinct early-response tasks; treating them as interchangeable tests of intrinsic tumor radiosensitivity would conflate different questions. Each assessment window answers only part of the question of durable control, and response after combined treatment reflects the regimen as a whole.

Serial imaging allows a model to incorporate observed treatment response, but the prediction must correspond to the intended decision point. A retrospective, multicenter study of 1039 NPC patients combined pre- and post-induction MRI with clinical factors in a graph-based model, reporting an external disease-free-survival concordance index (C-index) of 0.79 compared with 0.62 for tumor–node–metastasis (TNM) staging [70]. Because post-induction scans were inputs, this is a post-induction assessment rather than an initial-treatment predictor. Its reported outcome differences between concurrent-chemoradiotherapy subgroups remain nonrandomized comparisons, not proof that the model identifies who can omit concurrent treatment. A smaller MRI habitat study used 104 patients and a random training/test split to predict early response to concurrent chemoradiotherapy [71]. Spatial tumor subregions and longitudinal change may both characterize heterogeneity, but they address different outcomes and require different validation data.

Distant failure is a separate prediction target. In 102 NPC patients, a multi-kernel model combined contralateral parotid CT, MRI, and planned-dose features to classify distant metastasis after initial treatment [58]. Metastasis status was determined over follow-up averaging 36 months, not as a uniform 36-month event probability [58]. The full three-input combination favored the proposed classifier in the reported ablation comparison, whereas a comparator had higher AUCs for the three two-input combinations [58]. This result shows that performance depends on both input representation and learning method; it does not establish that fusion is uniformly superior. Because the analyzed region was a normal-tissue structure, its imaging and dose associations should not be interpreted as direct measurements of metastatic tumor biology; their incremental value over stage and EBV DNA also remains unestablished [58].

Nodal imaging further illustrates the distinction between phenotype recognition and treatment outcomes. An MRI study combining deep and radiomic features evaluated occult cervical nodal metastasis in 319 patients with early oral or oropharyngeal cancer, including external validation sets [72]. This is a diagnostic staging application, indirectly relevant to radiation-field decisions rather than direct evidence that elective irradiation can be omitted. By contrast, an AI pipeline for imaging-based extranodal extension in 397 HPV-positive oropharyngeal patients treated with upfront radiotherapy or chemoradiotherapy assessed both agreement with expert imaging classifications and subsequent outcomes [73]. The AI-defined phenotype was associated with worse overall and recurrence-free survival and distant control, while locoregional control was similar [73]. A nodal phenotype need not carry the same prognostic information for every failure pattern. Any use to alter local, regional or systemic treatment would need to address the relevant endpoint; a generic high-risk label is insufficient for that purpose.

Post-treatment surveillance introduces a further temporal distinction. A review of oropharyngeal squamous cell carcinoma (OPSCC) biomarkers identifies assay and threshold variability as important limitations for circulating tumor HPV DNA [74]. In the corrected analysis of 45 HPV-associated OPSCC patients treated with chemoradiotherapy, all four patients with two consecutively positive plasma tests developed biopsy-confirmed recurrence [75]. The small event count limits precision, and the repeat-testing rule should not be replaced by a single detectable result. Virus-independent HNSCC requires a different assay approach. A tumor-informed circulating tumor DNA (ctDNA) study reported successful testing in 100 of 116 patients, with insufficient tissue accounting for failures, and associated post-treatment positivity with poorer progression-free survival [76]. An individualized mutation/digital polymerase chain reaction (PCR) study monitored 18 of 26 enrolled patients after exclusions for absent tumor-specific mutations or assay-design limitations [77]. These studies support investigating molecular surveillance, but its apparent prognostic performance must be interpreted in light of assay availability and eligibility for testing.

During active treatment, longitudinal measurements can update risk rather than simply reproduce baseline risk categories. EP-SEASON prospectively followed 1000 NPC patients with circulating EBV DNA assessments at 11 time points during sequential chemotherapy and radiotherapy, identifying distinct trajectories and changing recurrence risks across treatment [78]. A post-radiotherapy analysis from an adjuvant-chemotherapy trial likewise associated subsequent EBV DNA clearance with better progression-free survival than persistent detectability [79]. Neither finding permits using an entire observed trajectory at an earlier decision time or establishes molecular clearance as a validated surrogate for an intervention’s survival effect. A combined imaging–blood strategy would need to specify when risk is updated, how discordant tests are handled and which action is triggered. Earlier detection or stronger prognostic separation alone leaves the benefit of additional treatment unproven.

### 5.2. Prognostic Biomarkers and Treatment-Predictive Biomarkers

A prognostic biomarker distinguishes outcome risks; a treatment-predictive biomarker must identify differences in the relative effects of specified treatments. In the 12-gene study, radiotherapy was associated with better survival in the low-score subgroup but not in the high-score subgroup [56]. Different subgroup significance levels, without a formal interaction test and adequate control of treatment selection, do not demonstrate effect modification or justify withholding radiotherapy. The ARTSCAN III analysis illustrates this distinction directly: despite prognostic differences within treatment arms, formal immune-score interactions comparing cisplatin–radiotherapy with cetuximab–radiotherapy were nonsignificant for local control and progression-free survival [66].

Formal interactions provide evidence about treatment-effect modification, but their validity depends on design and uncertainty. A three-gene radiosensitivity signature (RSS) showed adjusted score-by-radiotherapy interaction hazard ratios of 3.367 (95% CI, 1.323–8.565) in TCGA training data and 3.073 (95% CI, 1.004–9.406) in internal validation [20]. These are interaction terms on the reported score scale, not the treatment effect within a biomarker subgroup; nonrandom treatment allocation and the wide validation interval limit interpretation. Genomic-adjusted radiation dose (GARD) combines a transcriptomic radiosensitivity index with prescribed dose [80]. In a pan-cancer pooled analysis, its interaction with radiotherapy status was significant for overall survival (*p* = 0.011), but not first recurrence (*p* = 0.22); the head and neck NKI recurrence association was also nonsignificant [68]. Neither study prospectively tested a GARD- or RSS-guided head and neck treatment strategy.

Randomized trials of hypoxia modification provide a setting for biomarker evaluation. In archived DAHANCA 5 tumors, the 15-gene classifier showed an interaction with nimorazole for locoregional failure (*p* = 0.003): adjusted hazard ratios were 0.42 (95% CI, 0.25–0.68) in more-hypoxic and 0.98 (95% CI, 0.67–1.44) in less-hypoxic tumors [81]. This supports treatment-effect modification for that classifier and comparison, rather than merely different subgroup significance levels. NIMRAD instead evaluated a 26-gene signature in older or less-fit HNSCC patients unsuitable for concurrent platinum or cetuximab. Nimorazole did not significantly improve freedom from locoregional progression in the hypoxic group (adjusted HR, 0.72; 95% CI, 0.36–1.44); 36 failures occurred versus 66 anticipated in the revised design [82]. The different assays, populations, radiotherapy schedules, and statistical information preclude treating NIMRAD as an identical replication or collapsing the two studies into a universal hypoxia-treatment rule.

In NPC, post-radiotherapy EBV DNA identified a high-risk group, but adjuvant gemcitabine–cisplatin did not significantly improve relapse-free survival in the randomized comparison [83]. The subsequent clearance analysis arose from the same trial, not an independent replication [79]. By contrast, the NPC multi-omics study reported an IgA-marker interaction with induction chemotherapy, including validation in an independent trial-derived cohort for disease-free survival (*p* = 0.036), but not conventionally significant overall-survival interaction (*p* = 0.066) [46]. The full text identifies important qualifications: discovery-stage IgA thresholds were selected by interaction performance, and the trial protocols did not prespecify biomarker analyses. Thus, these results support a treatment-predictive candidate with independent trial-derived evidence, not prospective proof that assigning therapy by the marker improves outcomes, and not a rule for modifying radiation dose [46].

Recent transcriptomic signatures further illustrate the gap between prognostic modeling and radiation-specific inference. The six-gene cell death and cellular senescence index (CDCSI) and an immune-related prognostic model named consensus machine learning-driven prediction immunotherapy signature (CMPIS) were selected from 270 and 303 algorithm combinations, with reported across-cohort mean C-indices of 0.651 and 0.666, respectively [63,64]. These averages were used for model selection and therefore are neither independent test estimates nor a basis for ranking the two signatures [63,64]. Biological follow-up addressed different questions: CDCSI genes showed increased expression in cisplatin-selected FaDu/DDP cells, whereas high CMPIS and a separate high-risk transcriptomic group showed hypoxia and cell-cycle enrichment [63,64,65]. The former links a signature to a drug-selected cellular state; the latter links scores to expression programs that motivated radiation-related hypotheses. A change in clonogenic radiation response or a patient-level treatment interaction remains undemonstrated for both, so these findings contribute candidate biology and stop short of a dose-selection rule.

### 5.3. Transportability Across Cohorts, Regimens, and Data Domains

Paired molecular and imaging data illustrate both the rationale for integration and the constraints on its evaluation. In a positron emission tomography/computed tomography (PET/CT)–exome study, a combined marker incorporating CT histogram kurtosis and a cellular-senescence pathway state inferred from genomic data improved prognostic separation within the analyzed cohort [25]. The pathway label was a genomic inference, not a direct assay of cellular senescence. However, only 62 of 127 initially enrolled patients entered the paired analysis; missing or insufficient tissue for DNA extraction accounted for 59 exclusions [25]. Any performance increment must therefore be interpreted alongside modality availability. Unless a strategy for incomplete inputs is explicitly tested, a combined model can be evaluated only in patients who provide all required inputs.

Baseline MRI radiomics was evaluated in 136 patients with EBV-related NPC from non-endemic areas using ten-fold cross-validation. The combined clinical–radiomic model had an overall-survival C-index of 0.83 (95% CI, 0.75–0.91), compared with 0.78 (95% CI, 0.72–0.85) for the clinical model [84]. These estimates support within-cohort comparison, not independent external transportability. Geographic diversity alone cannot establish that a locked model transfers between populations. Assay availability also matters: insufficient tissue can prevent ctDNA testing or organoid establishment [39,76]. Studies should report patients from attempted testing through usable prediction, not only those with complete modalities.

Transportability concerns both where a model is tested and whether the treatment context remains comparable. The full-feature spatial proteomic model achieved a mean patient-level AUC of 0.754 in internal cross-validation of 31 cisplatin-based chemoradiotherapy patients, but only 0.483 when applied to 29 patients receiving mitomycin-based chemoradiotherapy in the same trial [32]. This failed transfer cannot by itself establish cisplatin-specific treatment prediction [32]. Across data cohorts, an RNA-derived immune score for oral squamous cell carcinoma showed lower three-year overall-survival discrimination in external Gene Expression Omnibus (GEO) datasets than in TCGA (AUC, 0.602 versus 0.731) [67]. These examples point to distinct limitations in transfer across regimens and cohorts; a common biological explanation for performance loss is not supported.

Performance differences are especially difficult to interpret when evaluation data help choose the model. An 18-gene NPC radiosensitivity score reported AUCs of 0.996 in development data and 0.823 in GSE32389, but the latter dataset participated in candidate-model comparison and selection [61]. The lower AUC therefore describes performance in reused evaluation data, not a fully independent estimate of transportability. The NBI study presents a different limitation: 267 images came from 57 patients, and the reported random image split did not establish patient separation [62]. This incomplete description indicates possible patient-level leakage, not proof that images from the same patient crossed partitions; nor can the external single-cell annotation substitute for external clinical evaluation of the image classifier [62].

Synthetic augmentation expands the training data, not the number of independent patients. In a postoperative MRI–dose study of 48 men, the training, validation, and test groups each contained 16 patients; synthetic expansion produced 160 training records from the original 16 cases [60]. Validation AUC was used to select the classifier, leaving only the small test group for subsequent evaluation [60]. The inputs also spanned different treatment states: MRI features represented the preoperative gross tumor volume, whereas dose features represented the postoperative planning target volume [60]. Their combination may encode complementary patient-level information, but reading it as spatial colocalization of the same tissue, or as a map of residual resistant clones, would overstate what was measured.

Validation claims must also be checked against the full analytical workflow. In a PET/CT–exome study, prognostic feature discovery preceded repeated cross-validation, raising a risk of outcome-informed feature selection across folds; the reported ordering does not, by itself, establish a confirmed implementation error [25]. A CT radiogenomic study instead used a random internal holdout from the same matched cohort; its targets were signaling-pathway alterations, not treatment response [85]. Data from multiple institutions therefore do not automatically constitute independent external validation, and predicting a pathway alteration does not establish a clinical treatment benefit. Clinical relevance depends on the endpoint, treatment comparison, and independence of evaluation; the number of molecular or imaging inputs is secondary.

## 6. Toxicity and the Dynamic Therapeutic Window

### 6.1. Normal-Tissue Risk and Functional Outcomes

A clinically meaningful toxicity model should address a measurable injury and an intervention capable of changing it. PARSPORT provides direct evidence for one such intervention: parotid-sparing IMRT reduced assessed grade 2 or worse xerostomia at 12 months compared with conventional radiotherapy and improved associated salivary and quality-of-life outcomes [6]. The joint guideline from the International Society of Oral Oncology (ISOO), Multinational Association of Supportive Care in Cancer (MASCC), and American Society of Clinical Oncology (ASCO) similarly supports tissue-sparing radiation modalities for prevention while distinguishing them from management of established salivary dysfunction [86]. These findings do not validate AI-guided patient selection, but they establish why dose to functional normal tissue is an essential comparator that cannot be omitted. The incremental value of molecular or imaging measurements should be evaluated beyond treatment exposure and symptoms already known at the proposed decision time.

Necrosis prediction in recurrent NPC concerns tolerance of re-irradiation, not toxicity during initial treatment of non-nasopharyngeal HNSCC. An MRI–dose fusion model reported an AUC of 0.81 in a 20-patient same-center holdout, versus 0.63 for MRI alone; its performance table did not provide confidence intervals [11]. A separate MRI signature achieved an external-cohort AUC of 0.756 (95% CI, 0.673–0.838) for one-year post-radiation nasopharyngeal necrosis [7]. Different dose representations, endpoint definitions, and validation designs prevent a direct ranking of these estimates.

Late swallowing symptoms require a distinct endpoint. A study in oropharyngeal and nasopharyngeal cancer used a one-time MD Anderson Dysphagia Inventory score of 60 or lower beyond 12 months after chemoradiotherapy, with 64 training and 23 test patients; only five test patients met the dysphagia definition [57]. This measures patient-perceived burden, not swallowing safety or symptom onset. Subjective assessments can miss silent aspiration [87]. Although clinical variables were considered, none entered the final imaging/dose models after selection, and model-guided improvement in swallowing outcomes was not tested [57]. Risk-directed supportive assessment should therefore be evaluated against its own functional objectives, which are distinct from any case for changing tumor dose.

More complex dose representation does not consistently improve prediction. A machine-learning study of severe acute oral mucositis found that models incorporating spatial dose information performed similarly to dose–volume-based models and still required external validation [88]. A separate prospective postoperative pilot in 36 patients combined normal tissue complication probability (NTCP) models with a blood-based phosphorylated ataxia telangiectasia mutated protein (pATM)-derived radiosensitivity assay and reported improved discrimination for acute mucositis and dysphagia [89]. The latter combines a functional assay with dose-based modeling, but its preliminary result does not establish a general host-radiosensitivity test or a benefit from changing treatment according to the assay. These studies favor direct comparisons of clinical/dose and biomarker-augmented models; neither finer spatial detail nor molecular testing should be assumed to add value. Acute mucosal injury, late swallowing dysfunction and necrosis should retain separate outcome definitions even when the same clinical workflow measures them.

The central treatment question is whether reducing predicted injury can preserve an acceptable probability of tumor control. A necrosis score developed among patients who all received re-irradiation estimates risk under observed care; it does not estimate the consequences of dose reduction, omission, or an alternative treatment [7]. Even an association between the score and mortality does not identify which treatment change would improve survival [7]. A decision-oriented evaluation should therefore compare tumor control and clinically important toxicity under the same specified treatment alternatives, while distinguishing injury-related death from other deaths and recurrence. This distinction matters when early death or preceding recurrence changes how a patient contributes to a fixed-time toxicity endpoint, as incompletely specified in the necrosis study [7]. Risk stratification provides a basis for testing a treatment strategy; whether high-risk patients should receive less treatment is a separate, unanswered question.

### 6.2. Dynamic Biofluid and Host Functional Signals

Serial sampling can describe treatment-associated changes without establishing an actionable prediction window. A prospective salivary-metabolomics study sampled nine men before radiotherapy, around 20 and 40 Gy, and shortly after completion; its higher-mucositis group comprised three patients with Common Terminology Criteria for Adverse Events (CTCAE) grade 2, not grade 3 or greater toxicity [69]. Pretreatment histidine and tyrosine showed exploratory associations with subsequent mucositis, without independent validation [69]. Evaluating adaptive use would require a predefined decision time and evaluation using only measurements available by that time; post-treatment samples cannot inform an earlier treatment choice.

On-treatment imaging offers a second route to tracking host-tissue change, but additional time points must add information beyond baseline risk and treatment exposure. In the dysphagia study, features from a synthetic CT derived from the final treatment-course cone-beam CT did not provide strong evidence of improvement over planning-CT models [57]. A radiomics review summarizes weekly parotid CT changes as a candidate xerostomia-monitoring signal [90]. The primary delta-radiomics study analyzed 68 patients and found that week-three parotid surface change added information to a pretreatment model based on mean gland dose and baseline xerostomia [91]. In another study of 31 patients and 59 parotids, radiomics/dosiomics models were evaluated alongside clinical and dose–volume comparators using nested cross-validation; the simpler comparators also performed strongly [92]. The gland count does not increase the independent patient denominator, and models using post-treatment images cannot be evaluated as if their information were available before radiotherapy. Serial measurements therefore require endpoint-specific assessment; later scans should not be assumed to be more informative. For an adaptive strategy, the useful window must leave time for the proposed action, and dose recalculated on a later anatomy should be distinguished from accumulated delivered dose.

Molecular sampling can complement these anatomical measurements, but interpretation still depends on specimen type and outcome timing. A prospective study with 47 evaluable baseline stool samples used shotgun metagenomics and found differences in gut microbial composition between patients with severe and lesser chemoradiotherapy-associated mucositis [93]. This is microbial sequencing evidence, unlike the oral keratinocyte functional assay discussed below, and it does not show that altering those taxa prevents mucositis. A larger plasma nuclear magnetic resonance (NMR) study sampled 302 head and neck cancer patients before, during and after treatment and associated metabolic or inflammatory measurements with swallowing and jaw-opening measures as well as clinical covariates [94]. Several functional associations were already present before treatment and should not be interpreted as predictions of newly induced radiation injury. Together with the small salivary study, these findings favor separating pre-existing host burden, treatment-associated change and future toxicity within an integrated model, with adjustment for exposure and baseline function.

Host functional assays provide a complementary baseline signal. Among 58 evaluable patients, pretreatment oral keratinocytes from those who subsequently developed World Health Organization (WHO) grade 3 mucositis showed reduced normalized spreading after ex vivo irradiation [8]. This measured normal-cell function, not microbial sequencing or an AI multi-omics predictor, and the study lacked validation and adjustment for mucosal dose and cisplatin exposure [8]. Cell culture and the nine-day spreading procedure also make turnaround time a practical concern for clinical use [8]. Both biofluid and functional signals therefore remain candidates for evaluation alongside treatment exposure and clinical risk; as established dose-adjustment rules they are premature.

## 7. From Model Output to Treatment Decisions

### 7.1. Risk of Bias, Standardization, Calibration, and Fairness

Technical credibility requires complementary assessments of study design, reporting and measurement consistency. The Transparent Reporting of a multivariable prediction model for Individual Prognosis Or Diagnosis guidance extended to AI (TRIPOD+AI) provides harmonized guidance for reporting the development and evaluation of regression and machine-learning prediction models [95]. The Prediction model Risk Of Bias ASsessment Tool extended to AI (PROBAST+AI) separates the quality of model development from the risk of bias in performance evaluation, examining participants and data sources, predictors, outcomes, and analysis [15]. The Image Biomarker Standardisation Initiative (IBSI) addresses a different requirement: reproducible radiomic computation, with 169 of 174 features standardized in its original study [19]. Agreement between software implementations supports measurement reproducibility, not the clinical validity of a head and neck radiotherapy model [19].

Calibration is essential when a predicted probability triggers a clinical action: accurate ranking does not ensure accurate absolute risk [16]. Evaluation should combine calibration curves with intercept and slope estimates, rather than treat a nonsignificant Hosmer–Lemeshow test as proof of calibration [16]. Performance and calibration should also be examined within clinically relevant populations, including viral and disease subtypes, with uncertainty acknowledged in small subgroups. PROBAST+AI incorporates fairness concerns, but neither a checklist nor an acceptable pooled metric establishes equitable performance across those populations [15].

Explainability characterizes a fitted model, not the effects of a biological intervention. In the seven-protein NPC study, Shapley additive explanations (SHAP) were used to attribute predictions to protein measurements, but conflicting textual descriptions of resistance and sensitivity require checking outcome coding before interpreting direction [59]. A feature attribution does not show that experimentally changing the protein alters radiation response. Mechanistic claims require perturbation evidence; patient-facing risk estimates additionally require calibration.

### 7.2. Independent Units and Complete Validation Pipelines

Validation should evaluate the complete prediction pipeline. Patients must be separated before data-adaptive preprocessing, with feature selection and tuning confined to training resamples; their images, cells, or serial measurements must not cross into evaluation data [15]. Internal holdouts, later same-center cohorts, external datasets used in model selection, and locked external tests are identified separately in Supplementary Table S2. Calibration, clinical-baseline comparison, and leakage concerns are recorded independently; unavailable information is not treated as evidence that a procedure was absent. Local model updating should be distinguished from evaluation of an unchanged model, and neither internal splitting nor multicenter provenance alone establishes transportability [20]. Documented reuse of evaluation data is distinguished from possible leakage when patient separation or preprocessing order is insufficiently described.

The distinction between external and independent evaluation is longstanding in normal-tissue modeling. An earlier review of head and neck NTCP studies found that external validation was uncommon and that most external evaluations were undertaken by the model developers, with only two independent external validation studies in its reviewed corpus [96]. Although this historical count does not cover all current studies, it illustrates why labels such as multicenter or externally tested are insufficient on their own. A useful evaluation must identify who performed the validation, whether the model and preprocessing were locked, and which patients were excluded because required assays or scans were unavailable. Performance after local revision should be reported separately from that of the transferred model to distinguish adaptation from unchanged transportability.

### 7.3. Clinical Context: Dose Reduction, Field Modification, and Drug Substitution

Randomized de-intensification studies show why favorable prognosis alone cannot justify treatment reduction. NRG-HN002 randomized 306 selected p16-positive OPSCC patients to 60 Gy IMRT with weekly cisplatin or accelerated IMRT alone [30]. Two-year progression-free survival (PFS) was 90.5% versus 87.6%; only the combination arm met both the historical PFS benchmark and swallowing criterion for phase III selection. The between-arm PFS hazard ratio was 0.67 (95% CI, 0.36–1.24), and acute grade 3–4 adverse events were more frequent with cisplatin [30]. This selection design neither establishes equivalence of 60 Gy to 70 Gy nor demonstrates a significant between-arm PFS difference. These trials inform treatment decisions but do not directly validate AI or multi-omics-guided allocation.

Drug substitution tested a different strategy. De-ESCALaTE HPV found no reduction in severe toxicity-event burden with cetuximab and worse two-year survival than with cisplatin (89.4% versus 97.5%; HR for cetuximab versus cisplatin, 5.0; 95% CI, 1.7–14.7) [17]. NRG/RTOG 1016 also failed cetuximab noninferiority; five-year survival was 77.9% (95% CI, 73.4–82.5) versus 84.6% (95% CI, 80.6–88.6) with cisplatin [18]. Overall moderate-to-severe toxicity proportions did not differ significantly, although acute grade 3–4 events per patient were fewer with cetuximab [18]. These distinct toxicity measures and trial designs should not be pooled. The results constrain the tested substitutions, not all biomarker-selected strategies, and neither trial tested AI-guided allocation.

Successful treatment reduction in another setting shows why these counterexamples cannot be generalized. In a randomized trial of 341 patients with low-risk stage II/T3N0 NPC, IMRT alone was noninferior to concurrent chemoradiotherapy for three-year failure-free survival and produced fewer grade 3–4 adverse events [97]. Eligibility excluded specified adverse nodal features and required plasma EBV DNA below 4000 copies/mL, so the result supports that defined clinical strategy rather than unselected omission of chemotherapy [97]. Conversely, in locoregionally advanced NPC, randomized evidence showed improved overall survival with gemcitabine–cisplatin induction before concurrent chemoradiotherapy [5]. The reported low-EBV subgroup comparison in that study should not be treated as proof of treatment interaction merely because its within-subgroup difference was nonsignificant. These trials support intensification or reduction under different eligibility criteria; such treatment selection need not be described as AI-enabled precision medicine.

Treatment volume, tumor dose, and systemic therapy are distinct components of treatment. In initially treated NPC, elective upper-neck rather than whole-neck irradiation met the trial’s regional-control noninferiority criterion [98]. A separate randomized report compared target delineation using pre- versus post-induction tumor extent and described lower late toxicity with the latter approach [99]; this is clinical context for volume adaptation, not evidence for a molecular dose-selection rule. In postoperative high-risk HNSCC, Japan Clinical Oncology Group (JCOG) trial JCOG1008 supported overall-survival noninferiority of weekly versus three-weekly cisplatin alongside radiotherapy [100]. None of these comparisons establishes interchangeability with recurrent NPC re-irradiation or validates an AI-guided treatment strategy.

### 7.4. Treatment-Effect Modification and Model-Guided Impact Studies

When a molecular score is claimed to identify differential relative treatment benefit, evaluation should test a prespecified score-by-treatment interaction for a defined treatment comparison. The RSS study provides an example of explicit interaction analysis, but its nonrandomized radiotherapy exposure and internal validation leave treatment-selection bias and transportability unresolved [20]. A confirmatory design should lock the assay, score, and analysis before evaluation in an independent treatment-comparison dataset, preferably with randomized allocation. An interaction for radiotherapy versus no radiotherapy would not by itself validate a choice between two radiation doses or concurrent drugs; conversely, selecting patients by absolute risk does not imply differential relative benefit and should be presented as an absolute-risk rationale rather than a predictive-biomarker claim.

Immunotherapy combinations make treatment context particularly important when interpreting molecular hypotheses. In molecularly unselected, locally advanced HNSCC, KEYNOTE-412 did not meet its prespecified event-free-survival significance threshold for pembrolizumab added to chemoradiotherapy: the reported *p* value of 0.043 did not cross the trial threshold of 0.024 [101]. By contrast, CONTINUUM reported improved event-free survival when sintilimab was added across induction, concurrent and adjuvant phases in selected high-risk, nonmetastatic NPC, with more grade 3–4 adverse events [102]. These findings do not constitute a randomized comparison of the trials, drugs or viral backgrounds. Differences in disease, eligibility and treatment schedule preclude attributing the contrast to a single immune feature. For an AI-derived immune or multi-omics signature, the next question is whether it modifies the effect of a specific tested regimen, not whether it resembles an immune phenotype associated with favorable outcomes elsewhere.

The intended treatment change also distinguishes different forms of AI-enabled adaptation. A radiomics review distinguishes adaptation to anatomical change while maintaining the prescribed target dose from response-adapted changes to dose based on radiosensitivity information [103]. A broader head and neck cancer AI review places contouring, dose-distribution prediction, and anatomy-responsive replanning within the treatment workflow [104]. These reviews support a distinction between delivery assistance and biological prescription selection. Reproducing contours or a planned dose distribution is a different task from establishing support for a lower tumor dose. Governance must reflect this distinction rather than treat every adaptive system as the same intervention. An earlier head and neck cancer big-data review proposed clinician-supervised algorithm refinement with updates tested in new patient cohorts [105]. In radiotherapy, updates should be documented as changes to measurement, risk estimation, or treatment recommendation and evaluated for that task before re-entering the clinical workflow. These workflow examples are contextual and are not counted as direct evidence of molecularly guided prescription benefit.

Clinical evaluation can also reveal a gap between dosimetric improvement and the intended patient benefit. DARTBOARD randomized 50 patients receiving definitive head and neck radiotherapy or chemoradiotherapy to daily adaptation with smaller planning margins or nonadaptive treatment with standard margins [106]. Although several gland doses were lower with adaptation, the trial did not demonstrate an improvement in its primary one-year patient-reported xerostomia endpoint [106]. The combined adaptation-and-margin intervention and small phase II sample limit broader interpretation; the result neither isolates the effect of an AI component nor rules out other adaptive strategies. Lower doses to selected organs therefore cannot substitute for measurement of the intended functional outcome.

Clinical impact concerns the entire model-guided strategy, including the action triggered by its threshold. A proposed evaluation should specify the comparator, decision time, acceptable efficacy loss if de-intensification is intended, and expected toxicity or functional benefit before patient assignment. Discrimination, calibration, and estimated net benefit can support evaluation, but do not establish that clinicians using the model improve patient outcomes [15,16]. A prospective impact study should therefore measure disease control and patient-relevant harms alongside test failures, turnaround time, treatment adherence, and additional workload; unavailable inputs should have a predefined fallback rather than silently exclude patients. Ongoing monitoring should assess subgroup performance and calibration drift, with any model update evaluated as a documented change [15,16]. Figure 1 summarizes these claim-specific requirements.

Reporting guidelines support transparent assessment at each stage of clinical evaluation. The Developmental and Exploratory Clinical Investigations of DEcision support systems driven by Artificial Intelligence guideline (DECIDE-AI) addresses early live clinical evaluation, including safety and human factors, before larger-scale studies [107]. The Standard Protocol Items: Recommendations for Interventional Trials extension for AI (SPIRIT-AI) concerns the protocol for an AI-intervention trial, requiring the planned system, workflow and input/output handling to be described [108]. The Consolidated Standards of Reporting Trials extension for AI (CONSORT-AI) addresses the resulting trial report, including human–AI interaction, required user skills and analysis of error cases [109]. These serve different purposes from prediction-model reporting and risk-of-bias assessment. Radiotherapy outcome evaluations should therefore document how clinicians review, override or act on model recommendations, rather than defer these questions until predictive accuracy has been established.

### 7.5. Limitations of This Review

This narrative review is subject to selection and citation bias. Saved queries and documented counts improve transparency, but targeted supplementation remains non-exhaustive; a complete intermediate screening denominator was not retained. There was no prospective registration or duplicate independent screening. The descriptive validation inventory is not a formal risk-of-bias assessment. All cited references were assessed in full text; details that a source did not report are labeled as unconfirmed rather than reconstructed. Evidence is concentrated in HNSCC and NPC, limiting extrapolation to less represented histologies and subsites. Viral background, initial versus recurrent disease, treatment exposure, endpoint definitions, and analytical units further restrict comparisons; in particular, recurrent NPC re-irradiation cannot stand in for treatment-naive HNSCC. Heterogeneity precluded quantitative pooling. 

## 8. Conclusions

Current evidence supports AI-assisted molecular characterization and outcome stratification more directly than model-guided treatment change in head and neck cancer. Joint-input integration, separate molecular analyses, and biological annotation contribute different kinds of information; adding modalities alone does not establish clinical value. Associations remain conditional on disease subtype, treatment, measurement time, and endpoint. Randomized trials support particular treatment decisions in defined populations, but they do not validate allocation by the AI models reviewed here. Independent validation and calibration are necessary foundations, not proof that acting on a prediction improves outcomes [15,16].

Future research should prospectively test predefined, locked model-guided strategies against appropriate care, measuring disease control, patient-relevant harm, and implementation burden. When differential relative benefit is claimed, the specified treatment interaction requires independent evaluation [20]. Missing inputs, model updating, and subgroup performance should be addressed before deployment. Such studies are a research priority, not an established benefit of current AI or multi-omics-guided radiotherapy.

## Figures and Tables

**Table 1:** Documented literature identification and selection stages. Counts refer to separate stages of a narrative review and do not represent a single systematic-review flow.

Stage	Number	Description
Initial database search	83	Thirty-eight records from Web of Science and 45 from PubMed.
After deduplication	59	Twenty-four duplicates removed; 59 titles/abstracts screened: 32 retained, 25 excluded, and 2 requiring clarification.
Initial full-text assessment	31	Thirty-two publications retained after screening; 31 available full texts assessed and subsequently cited. One publication had no readable full text and was not cited.
Earlier targeted supplementation	23	Twenty-three additional cited publications addressing specific biological, clinical, or methodological questions.
Targeted PubMed supplementation	55	Fifty-seven candidates assessed; 55 additional publications cited and 2 deferred.
Background and methodological references	Included above	Publications used for background, clinical context, or methodological explanation are included in the citation totals above, rather than counted as an additional set of core studies.
Total references cited	109	Thirty-one initial full-text publications + 23 earlier additions + 55 September 12 additions. All 109 cited references were assessed in full text.

**Table 2:** Selected primary studies grouped by inferential role: A, patient-level prediction and biomarker studies; B, functional or experimental investigations; and C, randomized treatment comparisons used as clinical context. Group A includes exploratory phenotypes, prognostic associations, and treatment-interaction analyses; these are not interchangeable evidence levels. The input column identifies joint inputs, parallel analyses, post hoc annotation, or a single measurement layer. Group C did not test allocation by the reviewed AI models. Secondary reviews and general background sources are not listed as primary evidence. Supplementary Table S2 applies a common descriptive validation framework. Population, treatment setting, endpoint, and prediction time remain study-specific.

Study and Evidence Type	Population and Setting	Inputs or Intervention	Endpoint/Analytical Target	Main Evidence	Validation and Interpretation Boundary
A|Gao et al. (2025) [11] Clinical prediction	Recurrent NPC; re-irradiation	Joint input: MRI–dose fusion. MRI and planned-dose features	Nasopharyngeal necrosis	Ninety-seven development and 20 same-center evaluation patients; 8 evaluation events; AUC 0.81 (CI not reported in the performance table).	Prognostic toxicity prediction; no treatment interaction or impact evaluation. Small evaluation set; imaging–dose fusion, not molecular multi-omics.
A|Ujjahan et al. (2025) [26] Clinical prediction with separate molecular analysis	Resectable locally advanced laryngeal cancer	Parallel analysis/post hoc annotation; no joint molecular predictor established. Radiomics; transcriptomic analysis	Response-adapted RT outcomes and radiosensitivity	Models incorporating follow-up imaging address a different prediction time from baseline-only models.	Outcome prediction and post hoc biology; joint molecular–imaging prediction not established. Matched patient-level joint molecular–imaging input is not established by the reviewed evidence.
A|Paetkau et al. (2024) [57] Clinical prediction	Oropharyngeal/NPC cohorts; curative chemoradiotherapy	Joint input: imaging–dose model; clinical candidates considered. Planning CT, dose and candidate clinical features	Patient-reported late dysphagia	Sixty-four training and 23 test patients; only 5 test patients had questionnaire-defined dysphagia. Final-course synthetic CT did not clearly improve prediction.	Prognostic symptom prediction; no model-guided impact evaluation. One-time MDADI score ≤60 at >12 months; not an instrumental test of swallowing safety or a clinical-impact study.
A|Grzeski et al. (2025) [32] Clinical biomarker prediction	HPV-negative locally advanced oropharyngeal or hypopharyngeal cancer	Single molecular layer: spatial proteomics. MALDI-MSI spatial proteomics	24-month NED versus recurrence or progression	Mean patient-level AUC 0.754 in 31 cisplatin-CRT patients; 0.483 on transfer to 29 mitomycin-CRT patients.	Outcome association and regimen transfer; no formal treatment interaction. Internal cross-validation and regimen transfer; failed transfer does not establish a treatment interaction.
A|Sheng et al. (2024) [58] Clinical prediction	One-hundred-and-two NPC patients after initial treatment	Joint input: CT–MRI–dose fusion. Contralateral parotid CT, MRI and planned-dose features	Distant metastasis	Fusion was evaluated by input-combination ablation; the full three-input model and two-input comparisons did not uniformly favor the same classifier.	Distant-metastasis prediction under observed care; no treatment interaction. Follow-up averaged 36 months, not a uniform 36-month risk endpoint; added value beyond stage and EBV DNA remains unestablished.
A|Lei et al. (2026) [59] Clinical prediction	NPC; 135 training and 164 validation patients	Single molecular layer: serum proteins. Seven-protein serum random-forest signature	Response 6 weeks after RT	Reported validation AUC 0.975.	Early combined-treatment response classification; no treatment-benefit interaction. The tumor-reduction and complete/non-complete response definitions are not fully reconciled; SHAP direction also requires outcome-coding checks.
A|Dong et al. (2025) [60] Clinical prediction	Forty-eight men with locally advanced hypopharyngeal cancer; postoperative CRT	Joint input: MRI–dose fusion across treatment states. Preoperative MRI and postoperative planned-dose features	Prognosis after postoperative CRT	16 patients each in training, validation and test groups; augmentation expanded 16 training cases to 160 records.	Prognostic association after postoperative CRT; no treatment interaction. Validation AUC informed model selection; synthetic records are not new patients, and MRI/dose regions represent different treatment states.
A|Li et al. (2025) [61] Transcriptomic classifier	NPC expression datasets	Single molecular layer: RNA score. 18-gene RNA score	Radiosensitivity label	AUC 0.996 in development and 0.823 in GSE32389.	Response-label classification; no clinical treatment-effect comparison. GSE32389 participated in candidate selection; its estimate is not an untouched external test.
A|Liu et al. (2023) [7] Clinical prediction	Recurrent NPC; re-irradiation	Post hoc RNA annotation of an MRI predictor. MRI risk score; post hoc transcriptomic annotation	Necrosis and survival	One-year necrosis AUC 0.756 (95% CI, 0.673–0.838) in the external cohort; outcome associations do not estimate the benefit of treatment change.	Prognostic toxicity/survival association; decision-curve analysis is not demonstrated impact. Transcriptomics did not enter the predictor; observed-care risk does not estimate benefit from dose reduction or treatment omission.
A|Tie et al. (2024) [62] Image classifier with biological annotation	Fifty-seven NPC patients; concurrent CRT	Post hoc biological annotation of an image classifier. Pretreatment NBI images; separate transcriptomic/cell annotation	MRI response within 3 days after CRT	Two-hundred-and-sixty-seven images supported classifier development and evaluation.	Early-response classification; no treatment interaction. Random image splitting did not establish patient separation; external biological annotation is not external clinical validation. Possible leakage is not confirmed overlap.
A|Luo and He (2026) [63] Prognostic modeling and cell experiments	HNSCC datasets; drug-selected FaDu/DDP cells	Parallel RNA modeling and cell experiments. Six-gene CDCSI	Prognosis; associated cellular biology	Across-cohort mean C-index 0.651 after comparing 270 algorithm combinations; genes were examined in cisplatin-selected cells.	Prognostic association; drug-selected cells do not establish irradiation response. Evaluation cohorts contributed to model selection; drug-selected expression is not clonogenic radiation-response evidence.
A|Yin et al. (2024) [64] Prognostic modeling	HNSCC datasets	RNA predictor with biological annotation. Immune-related CMPIS RNA signature	Prognosis and associated immune biology	Across-cohort mean C-index 0.666 after comparing 303 algorithm combinations; high-risk programs included hypoxia and cell-cycle enrichment.	Prognostic association; no established radiotherapy interaction. Model-selection performance is not an untouched test estimate; enrichment does not establish a RT interaction.
A|Luo et al. (2025) [65] Molecular discovery and prognostic modeling	HNSCC datasets	Joint multi-omics subtyping; separate RNA prognostic model. Multi-omics subtyping and prognostic modeling	Molecular subtypes and prognosis	A high-risk transcriptomic group showed hypoxia and cell-cycle enrichment.	Subtype/prognostic association; no established radiotherapy interaction. Prognostic and pathway associations do not establish differential radiation benefit.
A|Feng et al. (2026) [51] Prognostic and pathology modeling	HNSCC; includes an adjuvant-RT subgroup	Parallel RNA, pathology and IHC analyses. Lactate-related RNA score, digital pathology and IHC	Metabolic phenotype and survival	RNA risk was associated with poorer outcomes; external extreme pathology-prediction groups differed in LDHA/MCT1 staining.	Molecular-surrogate classification and prognostic association; not treatment-effect prediction. RNA and protein surrogates are not direct lactate measurements; phenotype classification differs from RT-outcome prediction.
A|Zhang et al. (2026) [36] Prognostic modeling and cell experiments	HNSCC datasets and CAL27 cells	Parallel single-cell, prognosis and perturbation analyses. Single-cell analysis, prognostic signature and ECT2 perturbation	Prognosis and tumor-growth biology	ECT2 knockdown reduced proliferation, DNA synthesis and colony formation.	Prognostic association and growth phenotype; no irradiation perturbation in the cited assays. The cited growth assays did not test changes in radiation response.
A|Haraldsson et al. (2026) [66] Clinical prediction and trial biomarker analysis	Oropharyngeal cancer; ARTSCAN III	CT predictor with tissue labels and an external RNA proxy; not joint predictor inputs. CT CNN; tissue CD8 phenotype and external RNA surrogate	Immune phenotype, local control, PFS and OS	Adjusted associations persisted for local control and PFS, but not OS; cisplatin–RT versus cetuximab–RT interactions were nonsignificant.	Prognostic association; formal interactions nonsignificant for local control and PFS. External proxy labels differ from tissue labels; prognostic separation does not establish treatment selection.
A|Essers et al. (2019) [35] Translational biomarker study	HNSCC; NKI CRT cohort and DESIGN comparison	Functional and molecular analyses; no joint patient-level multimodal predictor established. Drug-sensitivity/DNA-repair phenotype modeling	Survival and distant metastasis	Overall OS HR 1.53 (95% CI, 0.93–2.52); normoxic subgroup HR 2.83 (95% CI, 1.36–5.86).	Conditional prognostic association; no demonstrated treatment interaction. Conditional associations; the normoxic distant-metastasis association was not independently reproduced at statistical significance in DESIGN.
A|Diao et al. (2021) [67] Clinical biomarker modeling	Primary resectable oral SCC	Parallel IHC and RNA predictors. Separate seven-feature IHC SVM and RNA immune models	Survival and treatment-subgroup associations	IHC associations reproduced in testing/validation; RNA 3-year OS AUC 0.731 in TCGA versus 0.602 in external GEO datasets.	Prognostic association; observational treatment subgroups are not formal effect modification. Assay models are distinct; observational treatment subgroups do not establish effect modification.
A|Scott et al. (2021) [68] Pooled cohort treatment-interaction analysis	Pan-cancer pooled analysis; HNC subgroup	Joint input: RNA-derived RSI and radiation dose. Transcriptomic RSI and prescribed dose (GARD)	First recurrence and OS	Eleven cohorts, 7 cancers, 1615 patients; RT-status interaction for OS but not first recurrence.	Formal interaction for OS, not recurrence; no prospective model-guided clinical utility. HNC NKI recurrence association was nonsignificant; not a prospective dose-guidance trial.
A|Liu et al. (2020) [56] Prognostic modeling	Irradiated HNSCC in TCGA	Multi-omics-guided selection; final predictor is RNA-only. CNV/SNV and expression selection; final 12-gene RNA score	OS under observed treatment	Internal test AUC 0.661 at 3 years and 0.584 at 5 years.	Prognostic association; subgroup significance differences do not establish interaction. Feature preselection preceded splitting; nonrandom RT and subgroup significance differences do not establish interaction.
A|Sun et al. (2021) [20] Retrospective treatment-interaction modeling	HNSCC; TCGA training/internal validation	Single molecular layer: RNA signatures. RNA-derived RSI/immune stratification and three-gene RSS	OS; RSS-by-RT interaction	Adjusted RSS-by-RT interaction HR: 3.367 (95% CI, 1.323–8.565) in training and 3.073 (1.004–9.406) in internal validation; *p* = 0.011 and 0.049.	Formal score-by-RT interaction; internal validation and nonrandom treatment limit inference. Nonrandom RT allocation; internal validation, not independent treatment-guidance or impact validation.
A|Yatsuoka et al. (2021) [69] Exploratory longitudinal biomarker study	Nine men receiving head and neck RT	Single molecular layer: serial salivary metabolites. Serial salivary metabolomics	Treatment changes and mucositis	Samples before RT, around 20/40 Gy and after completion; the higher-mucositis group had 3 patients with grade 2 toxicity.	Treatment-associated changes and exploratory toxicity associations; no clinical utility evaluation. Exploratory baseline associations; no independent validation and no grade ≥3 endpoint in that group.
A|Li et al. (2026) [46] Multi-omics and trial biomarker study	Previously untreated locoregionally advanced NPC; discovery cohort and trial-derived tissue cohorts	Proteomic subtyping with cross-omics characterization and separate integrative analyses. Proteomic subtyping; cross-omics characterization; IgA tissue markers	Subtype biology and modification of systemic-treatment effects	Discovery: 240 patients, with assay-specific availability. Independent induction-chemotherapy cohort: DFS interaction *p* = 0.036; OS interaction *p* = 0.066.	Treatment-interaction evidence, not demonstrated marker-guided clinical utility. Discovery cutoff selection was outcome-informed; trial protocols did not prespecify biomarker analyses. Not a radiation-dose comparison.
B|Liu et al. (2024) [37] Experimental irradiation study	NPC experimental models	Functional perturbation; not a joint-input predictor. NAE1-related molecular work and MLN4924 perturbation	Tumor growth and irradiation response	Treated irradiated HNE1/HNE2 cells formed more surviving colonies; combined treatment produced larger tumors in an HNE1 xenograft model.	Functional irradiation response in models; not patient treatment interaction or clinical utility. Model-specific combination findings do not establish a clinical contraindication or a universal inhibitor effect.
B|Thomsen et al. (2025) [8] Prospective functional biomarker study	Head and neck cancer; 58 evaluable patients	Functional assay; not multi-omics integration. Pretreatment oral keratinocytes; ex vivo irradiation/spreading	Subsequent WHO grade 3 mucositis	Cells from patients with grade 3 mucositis showed reduced normalized spreading after irradiation.	Association with subsequent mucositis; no model-guided impact evaluation. Functional host assay, not microbial sequencing or AI fusion; no validation or adjustment for mucosal dose/cisplatin.
C|Yom et al. (2021) [30] Randomized phase II treatment comparison	Selected p16-positive OPSCC; 306 eligible patients	Not applicable: treatment intervention. Randomized 60-Gy IMRT with or without cisplatin	Two-year PFS and swallowing criterion	Two-year PFS 90.5% versus 87.6%; between-arm HR 0.67 (95% CI, 0.36–1.24). Only the combination arm met both phase III selection criteria.	Regimen-selection evidence against historical criteria; not biomarker interaction or AI utility. Historical-benchmark design; not equivalence of 60 Gy to standard 70 Gy, and not an AI-guided comparison.
C|Mehanna et al. (2019) [17] Randomized phase III treatment comparison	Low-risk HPV-positive OPSCC; De-ESCALaTE HPV	Not applicable: treatment intervention. Randomized cisplatin versus cetuximab with RT	Severe toxicity burden; tumor control	No reduction in severe toxicity-event burden; 2-year OS 97.5% with cisplatin versus 89.4% with cetuximab (HR for cetuximab, 5.0; 95% CI, 1.7–14.7).	Direct evidence on drug substitution; not an AI-guided strategy. Applies to this substitution strategy; aggregate toxicity does not imply identical adverse-event profiles.
C|Gillison et al. (2019) [18] Randomized phase III treatment comparison	HPV-positive OPSCC; NRG/RTOG 1016	Not applicable: treatment intervention. Randomized cisplatin versus cetuximab with RT	OS noninferiority and toxicity	Cetuximab failed OS noninferiority; 5-year OS 84.6% (95% CI, 80.6–88.6) with cisplatin versus 77.9% (73.4–82.5) with cetuximab.	Direct evidence on drug substitution; not an AI-guided strategy. Grade 3–4 toxicity proportions did not differ significantly, but acute events per patient were fewer with cetuximab; not biomarker-guided assignment.

Abbreviations: AI, artificial intelligence; AUC, area under the receiver operating characteristic curve; CI, confidence interval; CNV, copy-number variation; CNN, convolutional neural network; CRT, chemoradiotherapy; CT, computed tomography; EBV, Epstein–Barr virus; GARD, genomic-adjusted radiation dose; GEO, Gene Expression Omnibus; HNC, head and neck cancer; HNSCC, head and neck squamous cell carcinoma; HPV, human papillomavirus; HR, hazard ratio; IHC, immunohistochemistry; IMRT, intensity-modulated radiotherapy; MALDI-MSI, matrix-assisted laser desorption/ionization mass-spectrometry imaging; MDADI, MD Anderson Dysphagia Inventory; ML, machine learning; MRI, magnetic resonance imaging; NBI, narrow-band imaging; NED, no evidence of disease; NPC, nasopharyngeal carcinoma; OPSCC, oropharyngeal squamous cell carcinoma; OS, overall survival; PFS, progression-free survival; RSI, radiosensitivity index; RSS, radiosensitivity signature; RT, radiotherapy; SCC, squamous cell carcinoma; SHAP, Shapley additive explanations; SNV, single-nucleotide variation; SVM, support vector machine; TCGA, The Cancer Genome Atlas; WHO, World Health Organization. CDCSI, cell death and cellular senescence index; CMPIS, consensus machine learning-driven prediction immunotherapy signature; C-index, concordance index; CD8, cluster of differentiation 8; DNA, deoxyribonucleic acid; ECT2, epithelial cell transforming 2; LDHA, lactate dehydrogenase A; MCT1, monocarboxylate transporter 1; NAE1, NEDD8-activating enzyme E1 subunit 1; NKI, Netherlands Cancer Institute; RNA, ribonucleic acid; Gy, gray. Cell-line names, gene-expression accession numbers, and trial/cohort identifiers retain their source designations. DFS, disease-free survival; IgA, immunoglobulin A.

## Data Availability

No new data were created or analyzed in this study. Data sharing is not applicable to this article.

## Supplementary Materials

The following supporting information can be downloaded at https://www.mdpi.com/article/10.3390/biomedicines14102252/s1, Supplementary Methods S1, Literature identification and selection and Table S1. Comparison of integration methods; Table S2. Structured validation inventory.
