# Supplementary material for: Artificial Intelligence, Multi-Omics, and Multimodal Integration in Precision Radiotherapy for Head and Neck Cancer: From Biological Insights to Treatment Decisions
Source: Biomedicines. 2026 Oct 5;14(10):2252. doi: 10.3390/biomedicines14102252 (PMC13701673; doi:10.3390/biomedicines14102252)
Supplement: Supplementary file 1 [file biomedicines-14-02252-s001.zip › biomedicines-4591571-supplementary.pdf]

Supplementary Methods S1 Literature identification and selection

Initial searches were performed on 8 September 2026 in PubMed and Web of Science Core Collection. The saved moderate-scope queries below were selected for export. No date or language restriction appears in these query strings. The exports contained 45 and 38 records, respectively. Deduplication left 59 records; title/abstract screening retained 32, excluded 25, and left 2 requiring clarification. Of the retained publications, 31 had readable full texts and entered the cited initial set; one without readable full text was not cited.

Eligibility was determined by relevance to head and neck radiotherapy biology, disease control, treatment response, toxicity, or integration and validation. Diagnostic, surgical, or drug-target studies lacking a relevant radiotherapy connection were excluded from the initial core set. Targeted supplementation subsequently supplied relevant mechanisms, methods, clinical comparators, and background. It contributed 23 earlier citations and 55 additions on 12 September 2026. These roles overlap the retrieval stages: background references are included in the 109 total, not an additional set. This was not a prospectively registered systematic review, and screening was not independently duplicated. Additional relevant literature was identified through targeted supplementary searches and reference-list screening. The initial core search and supplementary context searches served different purposes; inclusion required relevance and a distinct contribution, not merely keyword matching.

PubMed query

("Head and Neck Neoplasms"[MeSH Terms] OR "Squamous Cell Carcinoma of Head and Neck"[MeSH Terms] OR "head and neck cancer"[Title/Abstract] OR "head and neck cancers"[Title/Abstract] OR HNSCC[Title/Abstract] OR "oral cancer"[Title/Abstract] OR "oropharyngeal cancer"[Title/Abstract] OR "nasopharyngeal carcinoma"[Title/Abstract] OR "laryngeal cancer"[Title/Abstract] OR "hypopharyngeal cancer"[Title/Abstract]) AND ("Radiotherapy"[MeSH Terms] OR radiotherap*[Title/Abstract] OR "radiation therapy"[Title/Abstract] OR chemoradiotherap*[Title/Abstract] OR irradiation[Title/Abstract] OR "adaptive radiotherapy"[Title/Abstract]) AND ("Multiomics"[MeSH Terms] OR multiomic*[Title/Abstract] OR "multi-omics"[Title/Abstract] OR "integrative omics"[Title/Abstract] OR "Single-Cell Analysis"[MeSH Terms] OR "Single-Cell Gene Expression Analysis"[MeSH Terms] OR "Spatial Transcriptomics"[MeSH Terms] OR "single-cell"[Title/Abstract] OR "single cell"[Title/Abstract] OR scRNA-seq[Title/Abstract] OR "spatial transcriptomics"[Title/Abstract] OR "spatial omics"[Title/Abstract] OR radiogenomic*[Title/Abstract] OR genomic*[Title/Abstract] OR transcriptomic*[Title/Abstract] OR proteomic*[Title/Abstract] OR metabolomic*[Title/Abstract] OR epigenomic*[Title/Abstract]) AND ("Artificial Intelligence"[MeSH Terms] OR "Machine Learning"[MeSH Terms] OR "Deep Learning"[MeSH Terms] OR "artificial intelligence"[Title/Abstract] OR "machine learning"[Title/Abstract] OR "deep learning"[Title/Abstract] OR "explainable AI"[Title/Abstract] OR "interpretable machine learning"[Title/Abstract] OR XAI[Title/Abstract] OR "neural network"[Title/Abstract] OR "neural networks"[Title/Abstract] OR "multimodal learning"[Title/Abstract] OR "multi-modal learning"[Title/Abstract])

Web of Science Core Collection query

TS=(("head and neck cancer*" OR "head and neck neoplasm*" OR HNSCC OR "oral cancer*" OR "oropharyngeal cancer*" OR "nasopharyngeal carcinoma*" OR "laryngeal cancer*" OR "hypopharyngeal cancer*") AND (radiotherap* OR "radiation therap*" OR chemoradiotherap* OR irradiation OR "adaptive radiotherapy") AND (multiomic* OR "multi-omic*" OR "integrative omic*" OR panomic* OR "single-cell" OR "single cell" OR scRNA-seq OR "spatial transcriptomic*" OR "spatial omic*" OR radiogenomic* OR genomic* OR transcriptomic* OR proteomic* OR metabolomic* OR epigenomic*) AND ("artificial intelligence" OR "machine learning" OR "deep learning" OR "explainable AI" OR "interpretable machine learning" OR XAI OR "neural network*" OR "multimodal learning" OR "multi-modal learning"))

Targeted PubMed supplementation on 12 September 2026

Targeted searches are as follows. The final assessed set comprised 57 candidates, of which 55 were cited and 2 deferred; it was not the complete database result set. The stored queries are reproduced below without implying exhaustive retrieval.

Topic query 1: ("head and neck cancer" OR "head and neck squamous cell carcinoma" OR "nasopharyngeal carcinoma" OR "oropharyngeal cancer") AND (radiotherapy OR chemoradiotherapy) AND (randomized OR meta-analysis OR guideline)

Topic query 2: ("head and neck" OR nasopharyngeal OR oropharyngeal) AND (radiotherapy OR radiosensitivity OR radioresistance) AND (genomic OR transcriptomic OR proteomic OR multiomics OR "multi-omics")

Topic query 3: ("head and neck" OR nasopharyngeal OR oropharyngeal) AND (radiogenomics OR radiomics OR "deep learning") AND (survival OR radiotherapy OR response)

Topic query 4: ("head and neck" OR nasopharyngeal OR oropharyngeal) AND ("circulating tumor DNA" OR "circulating HPV DNA" OR "Epstein-Barr virus DNA") AND (radiotherapy OR recurrence OR surveillance)

Topic query 5: ("head and neck" OR nasopharyngeal OR oropharyngeal) AND (radiotherapy) AND (dysphagia OR xerostomia OR mucositis) AND ("machine learning" OR "prediction model" OR randomized OR radiomics)

Topic query 6: ("head and neck squamous cell carcinoma" OR "nasopharyngeal carcinoma") AND ("single-cell" OR proteogenomic OR "spatial transcriptomics" OR hypoxia)

Topic query 7: ("multi-omics" AND (integration OR "machine learning")) OR "TRIPOD+AI" OR "DECIDE-AI" OR "CONSORT-AI" OR "SPIRIT-AI" OR "TRIPOD statement"

Descriptive study assessment

Table 2 records design, population, treatment, inputs, endpoint, independent patient counts and inferential role. Table S2 applies these common validation domains to all studies contributing substantially to patient-level prediction or methodological conclusions, and marks non-model studies as not applicable; unconfirmed information denotes details not reported in the source full text and is not coded as an absent procedure. All cited references were read in full text, and numerical and methodological claims were verified against full-text reports and available supplements. We did not assign a numerical quality score or claim formal, independently duplicated risk-of-bias assessment.

Table S1 Comparison of integration methods

Method	Objective and supervision	Input requirements	Missing data in the reported workflow	Interpretability	Principal limitations
DIABLO [23]	Supervised cross-omics feature selection and class discrimination	Matched sample-by-feature blocks plus class labels	The reported prediction scheme can vote using available blocks; this does not establish unrestricted missing-block training.	Sparse feature loadings and cross-block correlations	Outcome-driven selection requires nested validation; correlated features are not causal mechanisms.

Method	Objective and supervision	Input requirements	Missing data in the reported workflow	Interpretability	Principal limitations
MOFA / MOFA+ [22,27]	Unsupervised shared and assay-specific latent variation	Linked molecular views; MOFA+ also represents sample groups	Models partially observed entries and missing views for some samples, provided sufficient linked information remains.	Factor loadings, variance decomposition and enrichment	Factors may reflect batch or other variation; stability and downstream prediction require separate assessment.
MOGONET [24]	Supervised classification using omics-specific sample graphs	Aligned omics matrices, graph construction and class labels	Original evaluation used matched omics; missing-modality robustness should not be assumed from that evaluation.	Ranked features and omics-specific contributions	Graph construction, feature preselection and tuning require leakage control; classification is not treatment-effect estimation.
Similarity network fusion [28]	Unsupervised integration of sample-similarity networks	Comparable sample sets with a similarity network per data type	Original workflow includes preprocessing/imputation; the fusion step is not itself a general missing-modality model.	Integrated neighborhoods and subgroup structure	Depends on similarity and neighborhood choices; subtype separation does not establish clinical usefulness.
Weighted-nearest-neighbor analysis [29]	Unsupervised cell-level multimodal representation	Paired measurements of different modalities in the same cells	Modality weights accommodate varying informativeness; paired input is still required for the core workflow.	Cell-specific modality weights and integrated neighborhoods	Cell-level structure does not create independent patient samples or validate patient-level outcome prediction.

DIABLO, Data Integration Analysis for Biomarker discovery using Latent cOmponents; MOFA, Multi-Omics Factor Analysis; MOGONET, Multi-Omics Graph cOnvolutional NETworks. Missing-data support is implementation- and task-dependent; prediction with missing blocks does not imply valid training under every missingness pattern.

Table S2 Structured validation inventory

Study and task	Patient separation and external evaluation	Calibration	Selection and leakage considerations	Clinical or single-input comparison
Gao [11] Recurrent NPC necrosis	97 development / 20 same-center holdout patients; not independent-center validation.	Not identified in the consulted main report.	Small holdout; complete preprocessing independence needs clarification.	MRI-only and MRI–dose combinations; comprehensive clinical baseline not established.
Ujjahan [26] Laryngeal outcomes	Patient-based discovery split; additional response cohort. Molecular dataset is separate.	Not confirmed for the clinical prediction task.	Different imaging time points and response tasks must remain separate; joint molecular inputs not established.	Radiographic and clinical comparisons reported; task and timing differ.
Paetkau [57] Late dysphagia	64 training / 23 later same-center test patients; only 5 test events.	Not identified in the consulted main report.	Selection within cross-validation; authors report no significant blinding to test results.	Clinical candidates considered but absent from final models; imaging/dose combinations compared.
Grzeski [32] Post-CRT disease status	Patient-level cross-validation in 31 patients; transfer to 29 patients on another regimen.	Risk calibration not identified; mass-spectrometry calibration is a different issue.	Keep spectra grouped by patient; feature-selection scope and regimen shift matter.	Feature/model variants compared; incremental benefit over a clinical baseline not established.
Sheng [58] NPC distant metastasis	102 patients; independent external evaluation not established.	Risk calibration not identified in the consulted report.	Same patient data support input ablations; follow-up is not a uniform fixed-time risk horizon.	Input and classifier comparisons reported; stage/EBV-DNA baseline increment not established.
Lei [59] NPC early response	135 training / 164 validation patients; independence of the entire development pipeline requires clarification.	Training and validation calibration plots reported.	Multiple algorithms compared; response coding and feature-attribution direction require reconciliation.	Algorithms compared; added value over a clinical/EBV-DNA baseline not established.
Dong [60] Postoperative hypopharyngeal prognosis	48 patients: 16 training, 16 validation, 16 test; no external cohort.	Not identified in the consulted main report.	160 augmented training records are not 160 patients; validation AUC guided model choice.	Imaging/dose variants compared; robust clinical-baseline increment not established.
Li [61] NPC RNA response score	Separate expression datasets, but GSE32389 also informed candidate selection.	Not confirmed for the reported score.	Reused evaluation data are not an untouched external test.	Candidate algorithms compared; clinical-baseline increment not established.
Liu [7] Recurrent NPC necrosis	Separate patient cohorts, including an external center.	Calibration curve and goodness-of-fit assessment reported; not clinical impact.	Training-derived score/cutoff transferred; detailed preprocessing and endpoint limitations remain relevant.	Clinical/dose comparisons and decision-curve analysis reported.

Study and task	Patient separation and external evaluation	Calibration	Selection and leakage considerations	Clinical or single-input comparison
Tie [62]<br>NPC NBI response	57 patients / 267 images; random image splitting does not establish patient-disjoint sets.	Not identified in the consulted main report.	Random image splitting without established patient separation raises possible leakage; actual patient overlap is not demonstrated. Biological annotation is not an independent clinical test.	Image classifiers compared; clinical-baseline increment not established.
Luo and He [63]<br>CDCSI prognosis	Multiple patient cohorts; evaluation cohorts contributed to model selection.	Not confirmed for the retained model in this audit.	270 algorithm combinations ranked using across-cohort performance; not an untouched test estimate.	Clinical/prognostic comparisons cannot undo evaluation reuse.
Yin [64]<br>CMPIS prognosis	Multiple patient cohorts; evaluation performance informed model choice.	Nomogram calibration curve reported.	303 algorithm combinations compared; average C-index is selection performance.	Clinical characteristics and other signatures compared.
Luo [65]<br>Subtypes/prognosis	External patient datasets evaluated; average validation C-index used to choose the model.	Not confirmed in this audit.	Model selection used evaluation cohorts; subtype and prognosis tasks differ.	Clinical-variable nomogram reported; untouched clinical-baseline comparison not established.
Feng [51]<br>RNA/pathology phenotype	Patient-level pathology split; separate IHC cohort evaluates molecular-surrogate extremes.	Clinical risk calibration not confirmed.	Millions of patches do not increase patient N; IHC phenotype validation is not RT-outcome validation.	No established incremental RT-benefit model over clinical care.
Zhang [36]<br>Prognosis and ECT2	Patient datasets and CAL27 experiments; full pipeline independence not confirmed here.	Not confirmed in this audit.	Growth assays do not test irradiation response; do not convert biological follow-up into clinical validation.	No established treatment-benefit comparison.
Haraldsson [66]<br>CT immune phenotype	Patient cohorts: development, external molecular-proxy evaluation, and trial outcomes are distinct.	Authors state calibration was not feasible for the external proxy analysis.	Nested tuning reported for ML comparators; tissue versus RNA proxy labels introduce domain shift.	Radiomics comparison and adjusted clinical associations; treatment interactions nonsignificant.
Essers [35]<br>Repair phenotype/prognosis	NKI and DESIGN cohorts; subgroup replication differs by endpoint.	Absolute clinical risk calibration not confirmed.	Cell-derived predictions and patient subgroup associations are different analytical units.	Context-specific prognostic analyses; no randomized marker-guided treatment comparison.
Diao [67]<br>Oral SCC immune scores	IHC development/test/validation and external RNA datasets; assay models are separate.	Not confirmed for every model/endpoint in this audit.	Do not treat RNA and IHC models as direct assay transfer; observational treatment allocation persists.	Clinical prognostic analyses reported; no formal validated treatment-selection rule.
Liu [56]<br>12-gene OS signature	149 training / 138 internal test irradiated patients.	Nomogram calibration plots in training and test sets reported.	Outcome-related feature preselection preceded splitting; nonrandom RT comparison.	Clinical-variable nomogram reported; not a treatment-interaction analysis.

Study and task	Patient separation and external evaluation	Calibration	Selection and leakage considerations	Clinical or single-input comparison
Sun [20] RSS-by-RT interaction	236 training / 156 internal validation patients from TCGA; not external validation.	Absolute risk calibration not confirmed for the interaction claim.	Candidate-gene interaction selection and nonrandom RT exposure; wide interaction CI.	Clinical covariate adjustment and propensity matching; not prospective utility.
Spielvogel [25] PET/CT–exome prognosis	62 paired patients from 127 enrolled; repeated internal cross-validation.	Not confirmed in this audit.	Outcome-based feature discovery is described before resampling, raising possible selection leakage; the reported ordering alone does not prove an implementation error.	Combined and single-input markers assessed; selection must be nested for an unbiased increment.
Li [46] NPC subtyping and treatment markers	Discovery cohort of 240 with assay-specific availability; independent trial-derived tissue cohorts.	Treatment-marker analysis is not calibrated patient-risk prediction.	Discovery thresholds optimized on interactions; trial protocols lacked prespecified biomarker analyses. Subtype classifier is a separate task.	Treatment interactions and clinical covariates evaluated; no marker-guided impact trial.
Lan [72] Occult nodal diagnosis	319 patients from two centers; held-out and external diagnostic evaluations.	Nomogram calibration plot reported.	Diagnostic task in surgically assessed patients; no direct RT-omission outcome validation.	Radiomics versus combined deep/radiomic features compared.
Bologna [84] Non-endemic NPC prognosis	136 patients; ten-fold internal cross-validation, no independent external cohort.	Not identified in the consulted main report.	Normalization, selection and thresholding repeated within folds as described in the full text.	Clinical, volume, radiomic, and combined models compared.
Other contextual prediction reports [70,71,73,85,88,91,92]	Original setting and reported sample counts retained in the text; no uniform external-validation designation assigned.	Not uniformly verified across these reports.	These contextual citations were not given a complete study-level risk-of-bias rating; unconfirmed pipeline details remain unclassified.	Reported comparisons are not treated as demonstrated clinical utility.
Scott [68] Pooled GARD interaction	11 cohorts, 7 cancers, 1,615 patients; pan-cancer pooling with an HNC subgroup.	Not a calibrated individual-risk model evaluation.	Observed treatment and dose; pooled interaction does not establish a locked HNC dose-guidance strategy.	RT-status interaction for OS ($P = 0.011$), not recurrence ($P = 0.22$); no model-guided impact trial.
Yom [30] Randomized clinical context	306 eligible patients; randomized 60-Gy regimens. Model development/test splitting not applicable.	Not applicable: no prediction-model calibration.	Historical phase III selection benchmarks; not a 60-Gy versus 70-Gy equivalence test.	Two-year PFS 90.5% vs 87.6%; HR 0.67 (95% CI, 0.36–1.24). No AI-guided allocation.
Mehanna [17]; Gillison [18] Randomized clinical context	Separate randomized trials in defined HPV-positive oropharyngeal populations; not pooled model-validation cohorts.	Not applicable: no prediction-model calibration.	Drug-substitution comparisons; do not transfer results across trials or relabel as marker-guided allocation.	Survival and toxicity comparisons retained separately in Table 2 and Section 7.3.
Liu [37] Experimental irradiation	Cell and xenograft models, not independent patient-outcome cohorts.	Not applicable.	Functional perturbation evidence is restricted to the tested models and combinations.	Irradiated intervention/comparator experiments; no patient treatment-interaction claim.

Study and task	Patient separation and external evaluation	Calibration	Selection and leakage considerations	Clinical or single-input comparison
Yatsuoka [69] Exploratory metabolomics	9 patients with serial samples; 3 in the grade-2 mucositis group; no independent cohort.	Not established.	Repeated sampling does not increase patient N; no grade ≥3 endpoint in the stated subgroup.	Longitudinal and exploratory baseline associations, not an impact comparison.
Thomsen [8] Functional host assay	58 evaluable patients; cellular observations remain clustered within patients.	Not established.	Ex vivo irradiation assay; no adjustment for mucosal dose/cisplatin established in the reviewed analysis.	Association with later grade-3 mucositis, not validated model-guided benefit.

Not confirmed denotes information not reported in the source full text, not proof of absence; every cited reference was read in full text before this inventory was compiled. This is a descriptive inventory, not a formal risk-of-bias rating. AI, artificial intelligence; AUC, area under the receiver operating characteristic curve; CDCSI, cell death and cellular senescence index; CI, confidence interval; CMPIS, consensus machine learning-driven prediction immunotherapy signature; CRT, chemoradiotherapy; CT, computed tomography; EBV, Epstein–Barr virus; ECT2, epithelial cell transforming 2; HR, hazard ratio; IgA, immunoglobulin A; IHC, immunohistochemistry; ML, machine learning; MRI, magnetic resonance imaging; NBI, narrow-band imaging; NPC, nasopharyngeal carcinoma; OS, overall survival; PET, positron emission tomography; RNA, ribonucleic acid; RSS, radiosensitivity signature; RT, radiotherapy; SCC, squamous cell carcinoma; TCGA, The Cancer Genome Atlas; C-index, concordance index.
